# Excited-State Dynamics of Overlapped Optically-Allowed 1B_u_^+^ and Optically-Forbidden 1B_u_^−^ or 3A_g_^−^ Vibronic Levels of Carotenoids: Possible Roles in the Light-Harvesting Function

**DOI:** 10.3390/ijms11041888

**Published:** 2010-04-26

**Authors:** Yasushi Koyama, Yoshinori Kakitani, Takeshi Miki, Rebecca Christiana, Hiroyoshi Nagae

**Affiliations:** 1 Faculty of Science and Technology, Kwansei Gakuin University, Sanda, Hyogo 669-1337, Japan; 2 Kobe City University of Foreign Studies, Gakuen Higashimachi, Nishi-ku, Kobe 651-2187, Japan

**Keywords:** carotenoids, 1B_u_^+^, 3A_g_^−^, 1B_u_^−^ and 2A_g_^−^ states, diabatic (overlapped) vibronic levels

## Abstract

The unique excited-state properties of the overlapped (diabatic) optically-allowed 1B_u_^+^ and the optically-forbidden 1B_u_^−^ or 3A_g_^−^ vibronic levels close to conical intersection (‘the diabatic pair’) are summarized: Pump-probe spectroscopy after *selective* excitation with ∼100 fs pulses of all-*trans* carotenoids (Cars) in nonpolar solvent identified a symmetry selection rule in the diabatic electronic mixing and diabatic internal conversion, *i.e.*, ‘1B_u_^+^-to-1B_u_^−^ is allowed but 1B_u_^+^-to-3A_g_^−^ is forbidden’. On the other hand, pump-probe spectroscopy after *coherent* excitation with ∼30 fs of all-*trans* Cars in THF generated stimulated emission with quantum beat, consisting of the long-lived coherent diabatic cross term and a pair of short-lived incoherent terms.

## 1. Introduction

### 1.1. Low-Lying Singlet-Excited States and Their Light-Harvesting Function

*Energetics.* In bacterial photosynthetic systems having carotenoids (Cars) with *n* = 9–13 conjugated double bonds in the all-*trans* configuration, shorter-chain Cars (*n* = 9 and 10) are selectively bound to LH2 antenna complexes for the light-harvesting function, which includes the absorption of light energy followed by singlet-energy transfer to bacteriochlorophyll (BChl) [1]. The all-*trans* conjugated chain having *C*_2h_ symmetry gives rise to low-lying singlet states, including the *optically-allowed* 1B_u_^+^ and the *optically-forbidden* 2A_g_^−^, 1B_u_^−^ and 3A_g_^−^ states, concerning transitions from/to the ground 1A_g_^−^ state [2,3]. The 1B_u_^−^ and 3A_g_^−^ states of Cars were first identified by the measurement of resonance-Raman excitation profiles (RREP) [1].

**Figure 1.**
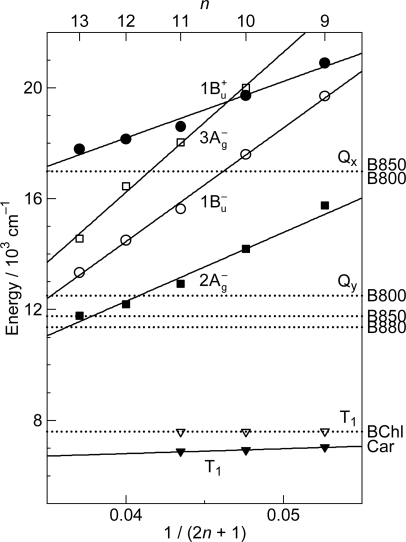
Energies of the 1B_u_^+^(0), 3A_g_^−^(0), 1B_u_^−^(0) and 2A_g_^−^(0) vibronic levels of Cars (*n* = 9–13) determined by measurement of resonance-Raman excitation profiles and those of the T_1_ state determined by emission spectroscopy of Cars (*n* = 9–11) bound to the LH2 complexes. For comparison, the energies of the Q*_x_* and Q*_y_* transitions and of the T_1_ state of BChls in LH2 and LH1 complexes are also shown (Reproduced from Ref. [1]).

**Figure 2.**
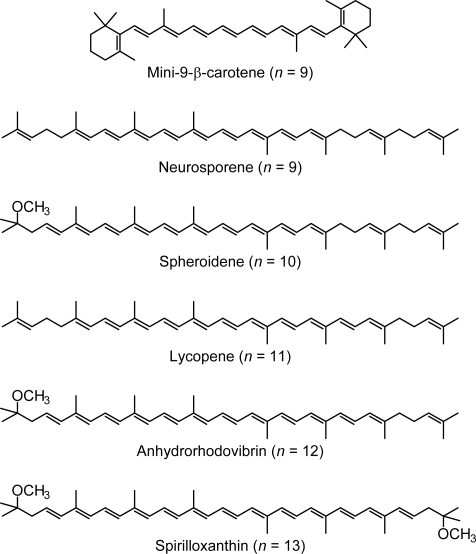
Chemical structures of typical Cars (*n* = 9–13) described in this summary.

As shown in Figure 1, the observed slopes of the linear relations for the 2A_g_^−^, 1B_u_^−^ and 3A_g_^−^ states, as functions of 1/(2*n* + 1), were in the ratio of 2:3.1:3.8, which is in excellent agreement with those theoretically predicted for shorter polyenes (*n* = 5–8) in the ratio of 2:3.1:3.7 [4]. This is actually the basis for the assignment of these forbidden electronic-excited states. Because of the set of linear relations, the next low-lying singlet state below the 1B_u_^+^ state is the 1B_u_^−^ state for the shorter-chain Cars (*n* = 9 and 10), whereas the 3A_g_^−^ state, for the longer-chain Cars (*n* = 11–13). Figure 2 shows the chemical structures of the relevant Cars (*n* = 9–13).

*Dynamics.* The singlet internal-conversion processes of 1B_u_^+^ → 1B_u_^−^ → 2A_g_^−^ → 1A_g_^−^ and the singlet-to-triplet fission followed by the triplet internal-conversion process of 1B_u_^−^ → T_2_ (A_g_) → T_1_ (B_u_) have been identified in the set of Cars (*n* = 9–13) in solution by pump-probe time-resolved spectroscopy using ∼100 fs pulses. The singlet internal conversion including the 3A_g_^−^ state, *i.e.*, 1B_u_^+^ → 3A_g_^−^ → 1B_u_^−^ → 2A_g_^−^ → 1A_g_^−^, has been identified in Cars (*n* = 11–13) by pump-probe spectroscopy using <5 fs pulses, and in Cars (*n* = 10 and 11) by subpicosecond time-resolved Raman spectroscopy using ∼100 fs pulses [1,5,6].

The low-lying singlet states of all-*trans* Cars have been found to give rise to plural channels of Car-to-BChl singlet-energy transfer during the processes of internal conversion in the order, 1B_u_^+^, 1B_u_^−^ and then 2A_g_^−^. This is the reason for the natural selection of the shorter-chain Cars in the all-*trans* configuration by antenna complexes. By transferring the rates of internal conversion within the Car and BChl *a* molecules in solution to those bound to the LH2 antenna complexes, the efficiencies of Car-to-BChl singlet-energy transfer through the 1B_u_^+^-to-Q*_x_*, 1B_u_^−^-to-Q*_x_* and 2A_g_^−^-to-Q*_y_* channels as well as the efficiencies of the 1B_u_^−^-to-T_1_ singlet-to-triplet fission reactions were determined [1,7,8]. The sums of efficiencies through the three channels for Cars (*n* = 9, 10, 11 and 11) were evaluated to be 88, 84, 51 and 54%, respectively, which nicely correlates to those determined by comparison of the electronic-absorption and fluorescence spectra, *i.e.*, 92, 89, 53 and 55%. The sudden decrease in the Car-to-BChl singlet energy-transfer efficiency, on going from *n* = 10 to *n* = 11, was explained by the closing of the latter two channels due to the lowering of the 1B_u_^−^ and 2A_g_^−^ energies shown in Figure 1. Thus, the important roles of the 1B_u_^−^ state in the singlet-to-triplet transformation and the Car-to-BChl singlet-energy transfer have been determined. However, the roles of the 3A_g_^−^ state in the light-harvesting function are left to be determined.

### 1.2. Diabatic Vibronic Levels

**Figure 3.**
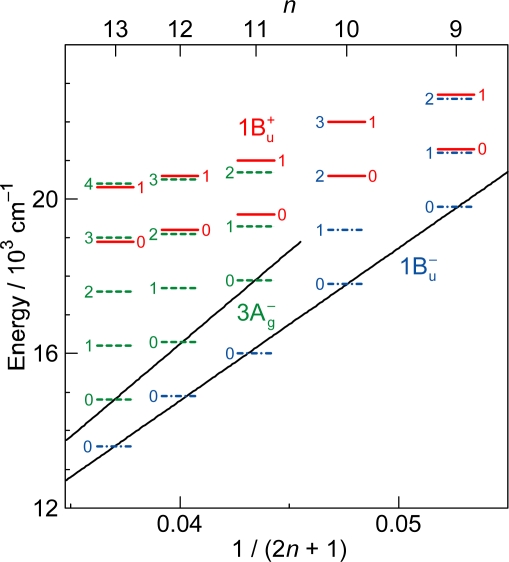
The vibrational ladder of the 1B_u_^+^ state (labeled on the right-hand-side) overlapped with those of the 1B_u_^−^ state in Cars (*n* = 9 and 10) and the 3A_g_^−^ state in Cars (*n* = 11–13) (both labeled on the left-hand-side). The spacing of all the vibrational ladders is set to be 1,400 cm^−1^ (Reproduced from Ref. [9]).

*Energetics.* Because of the unique linear relations among the 1B_u_^+^(0), 1B_u_^−^(0) and 3A_g_^−^(0) vibrational origins as shown in Figure 1, the 1B_u_^−^(0) level, for example, completely or approximately overlaps with the 1B_u_^−^(1) and 1B_u_^−^(2) levels in the shorter-chain Cars (*n* = 9 and 10), whereas with the 3A_g_^−^(1), 3A_g_^−^(2) and 3A_g_^−^(3) levels in the longer-chain Cars (*n* = 11, 12 and 13, respectively) as shown in Figure 3.

We will call the pair of overlapped levels ‘diabatic vibronic levels’ or ‘diabatic pair’, because a diabatic basis set, instead of an adiabatic basis set, becomes necessary to theoretically describe their excited-state properties [10] (see Section 1.3). Thus, the shorter-chain Cars can form the 1B_u_^+^ + 1B_u_^−^ diabatic pairs, whereas the longer-chain Cars, the 1B_u_^+^ + 3A_g_^−^ diabatic pairs. It is to be noted that the energy gap between the diabatic pair is the largest in Car (*n* = 11) and negligible in Car (*n* = 10) (Figure 3), if we assume the interval of vibronic levels to be ∼1,400 cm^−1^, inclusively taking into account the C=C (ν_1_) and C–C (ν_2_) stretching modes.

**Figure 4.**
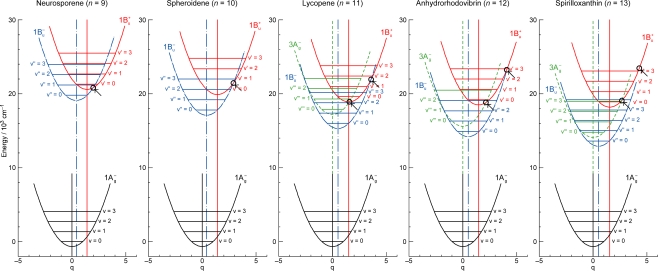
The 1B_u_^+^, 1B_u_^−^ and 3A_g_^−^ potentials and conical intersections between the 1B_u_^+^ and 1B_u_^−^ potentials in Cars (*n* = 9 and 10) and between the 1B_u_^+^ and 3A_g_^−^ or 1B_u_^−^ potentials in Cars (*n* = 11–13). Those conical intersections relevant to diabatic electronic mixing and diabatic internal conversion are indicated.

The definition of the diabatic pair includes *not only* that the pair of vibronic levels is overlapped with each other, *but also* that the overlapped vibronic levels are located close to the conical intersection (see Figure 4). Concerning the set of potential functions, the shifts of the 1B_u_^+^, 1B_u_^−^ and 2A_g_^−^ potential minima, in reference to the ground 1A_g_^−^ potential minimum, were determined by the Franck-Condon simulations of stationary-state fluorescence spectra from Cars (*n* =9–13) [9]. The 3A_g_^−^ potential has been determined by pump-probe stimulated-emission spectroscopy of Cars (*n* = 11–13) after coherent excitation using 30 fs pulses [11], the details of which will be described in Section 3.1 of this article. Figure 4 clearly shows that the above-mentioned diabatic pairs, *i.e.*, 1B_u_^+^(0) + 1B_u_^−^(1) and 1B_u_^+^(0) + 1B_u_^−^(2) in Cars (*n* = 9 and 10) as well as 1B_u_^+^(0) + 3A_g_^−^(1), 1B_u_^+^(0) + 3A_g_^−^(2) and 1B_u_^+^(0) + 3A_g_^−^(3) in Cars (*n* = 11, 12 and 13), are located close enough to the conical intersection, and satisfies the above condition of the diabatic pair. Another set of five pairs, which are located one vibrational-quantum higher, also fits this definition.

*Dynamics.* The all-*trans* conjugated chain of Cars has approximate *C*_2h_ symmetry in the ground state and, as a result, the singlet electronic states can be classified by symmetry into *k*A_g_^−^, *l*B_u_^−^, *m*B_u_^+^ and *n*A_g_^+^, where the + and – signs are called Pariser’s labels [2] and *k*, *l*, *m* and *n* indicate the ordering of the electronic states having the same symmetry (from the lowest to the higher energies). As shown in Figure 1, the low-lying singlet-excited states are in the order, 2A_g_^−^, 1B_u_^−^, 3A_g_^−^ and 1B_u_^+^. Concerning Pariser’s labels, optical transitions are allowed (forbidden) between electronic states with different signs (the same sign), whereas internal conversion is allowed (forbidden) between electronic states with the same sign (different signs).

As will be described in the next section, both the optically-allowed 1B_u_^+^ counterpart and the optically-forbidden 1B_u_^−^ or 3A_g_^−^ counterpart keep their own symmetry properties, and behave as if they were totally independent symmetry-wise even after forming the diabatic pair.

### 1.3. Conical Intersection and Conservation of Symmetries in Singlet-Excited States

The unique characteristics of the low-lying singlet-excited states of Cars in the all-*trans* configuration can be summarized as follows: (a) Singlet-excited states having different symmetries, energetically in the order of 1B_u_^+^, 3A_g_^−^, 1B_u_^−^ and 2A_g_^−^, are closely located within a small energy difference in the region of 1–3 vibrational quanta (see Figure 1). (b) The minima of the 3A_g_^−^, 1B_u_^−^, 1B_u_^+^ and 2A_g_^−^ potentials are located, in this order, in a small region of mass-adjusted normal coordinate of *q* = 0–2 (see Figure 4). (c) A pair of excited-state potentials with different symmetries can cross at the conical intersection keeping their own symmetry characteristics, the details of which are described below.

In the vicinity of a conical intersection, the Born-Oppenheimer approximation - on which the adiabatic description is based - breaks down. The reason for this is as follows: (1) The derivative coupling can be expressed as the vibronic coupling divided by the potential-energy difference like

(1) $$\langle \varphi_{i}^{A}|\frac{\partial }{{\partial Q}_{a}}|\varphi_{j}^{A}\rangle \;=\;\langle \varphi_{i}^{A}|\frac{\partial H}{{\partial Q}_{a}}|\varphi_{j}^{A}\rangle /\left\{V_{ii}(Q)-V_{j\;j}(Q)\right\}$$

In the complete all-*trans* planar configuration in the ground state having the *C*_2h_ symmetry, the vibronic-coupling term between a pair of electronic states (*φ_i_* and *φ_j_*) having different Pariser’s ± labels vanishes. However, when the Car molecule is excited to the 1B_u_^+^ state, for example, there is a good chance for the conjugated chain to take a twisted conformation (degrading the *C*_2h_ symmetry) and to give rise to a certain value of the vibronic coupling. Then, in the vicinity of conical intersection where *V_ii_*(**Q**) – *V_jj_*(**Q**) approaches to 0, the derivative coupling diverges. (2) The time-dependent perturbation theory shows that the rate of change in any of the MO and CI coefficients is proportional to the value of *V_ii_*(**Q**) – *V_jj_*(**Q**) and, therefore, the reorganization of electronic wavefunction practically cannot take place in the vicinity of the conical intersection.

Therefore, it becomes absolutely necessary to use diabatic expression, instead, setting the derivative coupling to be zero, *i.e.*,

(2) $$\langle \varphi_{i}^{D}|\frac{\partial }{{\partial Q}_{a}}|\varphi_{j}^{D}\rangle =0$$

and fix the MO and CI coefficients by the use of the orthogonal floating atomic orbitals. As a result, a pair of electronic wavefunctions, expressed by such a diabatic basis set, keeps the symmetry of each electronic state at any nuclear coordinate (**Q**), which makes the pair of nuclear potentials having different symmetries cross each other at the conical intersection, as shown in Figure 4.

The above consideration has rationalized the apparently unique characteristics of the diabatic pair of electronic states: At the first glance, it looked strange and accidental that the symmetry properties of singlet-excited states conserve, as if they were totally independent from each other. However, it has turned out to be quite logical after we carefully consider the characteristics of the diabatic pair.

Working on the set of Cars (*n* = 9–13) has been very fortunate, because the shorter-chain Cars (*n* = 9 and 10) and the longer-chain Cars (*n* = 11–13) form completely different diabatic pairs in symmetries, *i.e.*, 1B_u_^+^ + 1B_u_^−^ and 1B_u_^+^ + 3A_g_^−^, respectively. This situation has enabled us to make a comparison between the two different combinations of symmetries in these diabatic pairs, and to establish the symmetry notation of the relevant singlet-excited states we have proposed.

In the electronic mixing of the diabatic pair (‘diabatic electronic mixing’) as well as in the internal conversion from a diabatic pair (‘diabatic internal conversion’), we have found a common symmetry selection rule, *i.e.*, ‘1B_u_^+^-to-1B_u_^−^ is allowed but 1B_u_^+^-to-3A_g_^−^ is forbidden’. When a 1B_u_^+^ + 1B_u_^−^ diabatic pair vibrationally relaxes down to the bottom of the 1B_u_^+^ potential, for example, the 1B_u_^+^(0) optically-allowed counterpart relaxes through radiative transition to the 2A_g_^−^ or 1A_g_^−^ state, whereas the 1B_u_^−^ optically-forbidden counterpart relaxes through internal conversion to the iso-energetic 2A_g_^−^ vibronic level followed by vibrational relaxation in the particular manifold.

The above experimental results (to be described in detail in Section 2.2) evidence that the symmetry of each electronic state is totally conserved during the formation of the diabatic pair as well as in the splitting of the diabatic pair into the optically-allowed and the optically-forbidden counterparts.

### 1.4. Time-Resolved Spectroscopies Used

**Figure 5.**
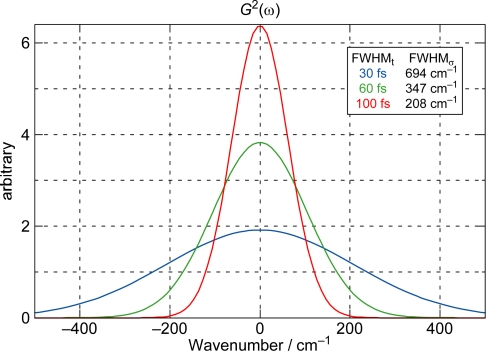
Intensity profiles as well as numerical correlations (inset) between the time duration (FWHM_t_) and the spectral width (FWHM_σ_) for the 30, 60 and 100 fs pulses.

*Pump-probe stimulated-emission and electronic-absorption spectroscopy.* We used both ∼100 fs and ∼30 fs pulses for pump-probe stimulated-emission and electronic-absorption spectroscopy. Correlation between the time-duration and the spectral-width of these pulses is presented in Figure 5, showing the intensity profiles and the numerical values of FWHM.

In comparison to the energy gap between the diabatic pair of Car (*n* = 11) (∼300 cm^−1^) shown in Figure 3, for example, the ∼100 fs pulses with the FWHM_σ_ of ∼200 cm^−1^ still tend to *selectively* excite one of the counterparts of the diabatic pair, whereas the ∼30 fs pulses with the FWHM_σ_ of ∼700 cm^−1^ can excite the optically-allowed and optically-forbidden diabatic counterparts *simultaneously* and *coherently*. Therefore, we call excitation with ∼100 fs pulses ‘selective excitation’, whereas excitation with ∼30 fs pulses ‘coherent excitation’.

Visible-pump and near infrared-probe spectroscopy *mainly* probes transient-absorption spectra with some contribution of stimulated emission, while visible-pump and visible-probe spectroscopy probes the strong 1B_u_^+^ stimulated emission as the optically-allowed counterpart. The latter spectroscopy is useful to identify stimulated emission from the optically-forbidden 1B_u_^−^ or 3A_g_^−^ counterpart of the diabatic pair, if any, and to conclude the presence or absence of the diabatic electronic mixing.

*Kerr-gate fluorescence spectroscopy.* We used this technique to probe fluorescence (mainly spontaneous emission) from the optically-forbidden vibronic levels of the Car molecules when they are being *vibronically* excited. This spectroscopic technique directly determines the energies of the emitting vibronic levels. Further, the excited-state molecules are free from disturbance by the probing radiation.

### 1.5. Contents of This Review

After the Introduction (Section 1), we are going to correlate the unique excited-state properties of the diabatic vibronic levels that we have found most recently. Those findings are classified into different categories in terms of the spectroscopic techniques used:

Section 2: We used ∼100 fs pulses for selective excitation: First, we excited to the 1B_u_^+^(0) level of a set of all-*trans* Cars (*n* = 9–13) in nonpolar solvent, and mainly probed transient absorptions by the use of near-infrared (NIR) white continuum to determine the 1B_u_^+^, 1B_u_^−^ and 3A_g_^−^ lifetimes (Section 2.1). Then, we excited the same set of Cars to the 1B_u_^+^(0) level and examined the presence or absence of stimulated emission from the optically-forbidden counterpart by the use of visible (VIS) white continuum. As a result, we found a symmetry selection rule in the diabatic mixing and diabatic internal conversion (Section 2.2). We also examined all-*trans*- and 15-*cis*-*β*-carotenes in nonpolar and polar solvents, and tried to find the effects of *cis*–*trans* configurations and those of the polarization of the conjugated chain by polar solvents on the initial stimulated emission patterns. The effect of aggregation was also found (Section 2.3).

Section 3: We used ∼30 fs pulses for coherent excitation of Cars (*n* = 11–13) in polar solvent, THF. Here, we observed stimulated emission followed by transient absorption from the 3A_g_^−^ counterpart, exhibiting a single peak with the 3A_g_^−^(0) energy. The results lead us to conclude that the shift of the 3A_g_^−^ potential, with respect to the ground 1A_g_^−^ potential, is negligible (Section 3.1). The stimulated emission after coherent excitation of Cars (*n* = 9 and 10) actually consisted of three components; one from the long-lived 1B_u_^+^ + 1B_u_^−^ diabatic pair, and the other two from the short-lived 1B_u_^+^ and 1B_u_^−^ counterparts. On the other hand, the stimulated emission after coherent excitation of Car (*n* = 11–13) consisted of one, from the long-lived 1B_u_^+^ + 3A_g_^−^ diabatic pair and the other two, from the short-lived 1B_u_^+^ and 3A_g_^−^ counterparts. The set of three components was explained by the mechanisms of quantum beat (Section 3.2). The same type of stimulated emission consisting of three components was observed after coherent excitation of Cars (*n* = 9–11) bound to the LH2 antenna complexes, which accompanied the shift of the 1B_u_^−^(0) and 3A_g_^−^(0) levels to the lower energies and efficient triplet generation (Section 3.3).

Section 4: We used ∼100 fs pulses to excite Cars (*n* = 9–12) to the 1B_u_^+^(3) or 1B_u_^+^(4) level and probed fluorescence, by Kerr-gate fluorescence spectroscopy, to examine the slowest two steps of vibrational relaxation, *i.e.*, 1B_u_^+^(2) → 1B_u_^+^(1) → 1B_u_^+^(0). We found the breakdown of the above-mentioned symmetry selection rule in the diabatic electronic mixing and diabatic internal conversion, due to the degradation of molecular symmetry, while the Car molecules were being excited (Section 4.1).

Section 5: We will summarize the results obtained (Section 2–Section 4) and discuss the future trend of the present line of research.

After Section 6: Conclusion, we will introduce Section 7: Relevant work done by other investigators.

## 2. Pump-Probe Spectroscopy: Selective excitation

### 2.1. Excitation to the 1B_u_^+^(0) Level of Cars (n = 9–13) in Nonpolar Solvent and Probing in the NIR Region [12]

As shown in Figure 1, the next low-lying singlet state below the 1B_u_^+^ state is the 1B_u_^−^ state in Cars (*n* = 9 and 10), whereas it is the 3A_g_^−^ state in Cars (*n* = 11–13); further, in Cars (*n* = 10), the 1B_u_^+^ state is overlapped with the 3A_g_^−^ state. Since the set of Cars is dissolved in nonpolar solvent (*n*-hexane or a mixture of *n*-hexane and benzene), the conjugated chains are expected to keep the *C*_2h_ symmetry in the ground state, before selective excitation with ∼100 fs pulses (the same symmetry should be conserved immediately after excitation).

**Figure 6.**
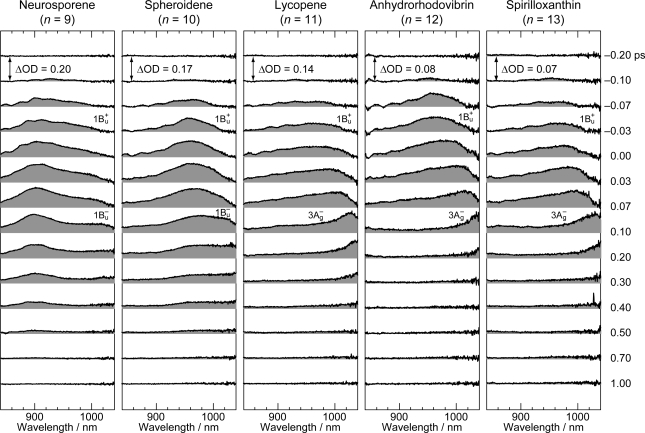
Subpicosecond time-resolved spectra after excitation with ∼100 fs pulses to the 1B_u_^+^(0) level of Cars (*n* = 9–13) in nonpolar solvent probed in the NIR region (Reproduced from Ref. [12]).

**Figure 7.**
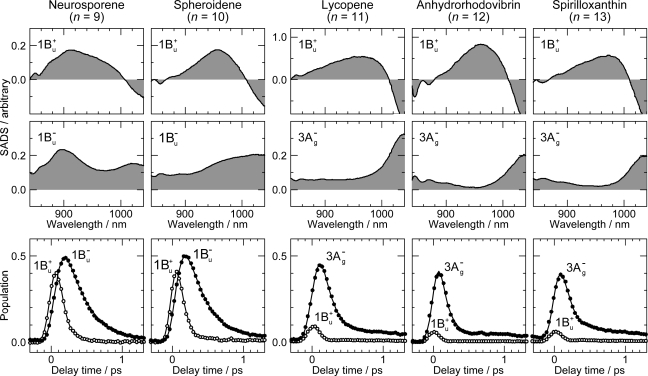
Species-associated difference spectra (SADS) and time-dependent changes in population for the 1B_u_^+^ and 1B_u_^−^ states of Cars (*n* = 9 and 10) and for the 1B_u_^+^ and 3A_g_^−^ states for Cars (*n* = 11–13) obtained by singular-value decomposition (SVD) followed by global fitting (Reproduced from Ref. [12]).

Figure 6 exhibits the pump-probe time-resolved spectra of Cars (*n* = 9–13), and Figure 7 shows the species-associated difference spectra (SADS, top) and time-dependent changes in population (bottom) that have been obtained by singular-value decomposition (SVD) followed by global fitting of the spectral-data matrices by the use of a two-component sequential model. The first component, appearing immediately after excitation, is ascribable to the optically-allowed 1B_u_^+^ state to which all the Car molecules were excited by the absorption of photons; each SADS consists of transient absorption and stimulated emission. The second component, appearing around 0.2 ps after excitation, is ascribable to the 1B_u_^−^ state in the shorter-chain Cars (*n* = 9 and 10), whereas to the 3A_g_^−^ state in the longer-chain Cars (*n* = 11–13), according to the energy diagram (Figure 1). The contribution of the 3A_g_^−^ transient absorption is also seen in the second component of Car (*n* = 10) as expected.

The decay time constants listed in Table 1 show that the 1B_u_^+^ lifetimes of Cars (*n* = 9 and 10) are on the order of 0.1 ps, which is much longer than those of Cars (*n* = 11–13) on the order of 0.01 ps. On the other hand, the 1B_u_^−^ lifetimes are around 0.25 ps, whereas the 3A_g_^−^ lifetimes are around 0.10 ps.

### 2.2. Excitation to the 1B_u_^+^(0) and 1B_u_^+^(1) Levels of Cars (n = 9–13) in Nonpolar Solvent and Probing in the VIS Region [10]

**Figure 8.**
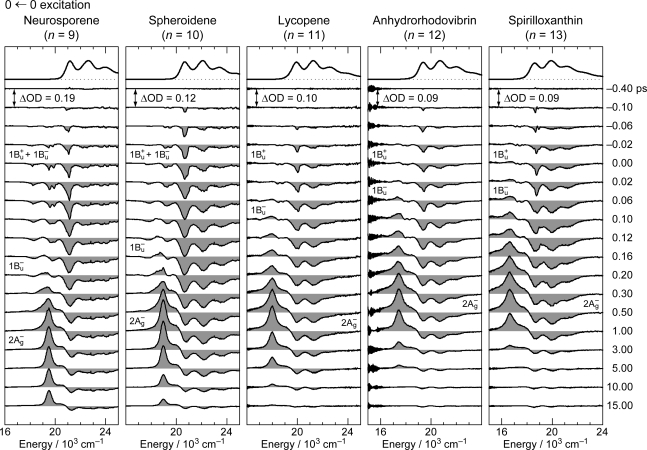
Subpicosecond time-resolved spectra after excitation with ∼100 fs pulses to the 1B_u_^+^(0) level of Cars (*n* = 9–13) in nonpolar solvent probed in the visible region (Reproduced from Ref. [10]).

Figure 8 shows the pump-probe time-resolved spectra of Cars (*n* = 9–13) in nonpolar solvent after selective excitation with ∼100 fs pulses to the 1B_u_^+^(0) level (called ‘0 ← 0 excitation’). Here, we can see the singlet-state internal-conversion processes of 1B_u_^+^ → 1B_u_^−^ → 2A_g_^−^ → 1A_g_^−^ (ground). In this subsection, we focus on the *initial* stimulated emission patterns that are useful in examining the presence or absence of diabatic electronic mixing between the optically-allowed 1B_u_^+^(0) and the iso-energetic optically-forbidden 1B_u_^−^ or 3A_g_^−^ vibronic levels. In this relation, we notice that the intensity of the strongest stimulated emission peak relative to the second-strongest one is much higher in the shorter-chain Cars (*n* = 9 and 10) than in the longer-chain Cars (*n* = 11–13). The higher intensity in the former has turned out to be due to additional contribution of the 1B_u_^−^ stimulated emission (*vide infra*).

**Figure 9.**
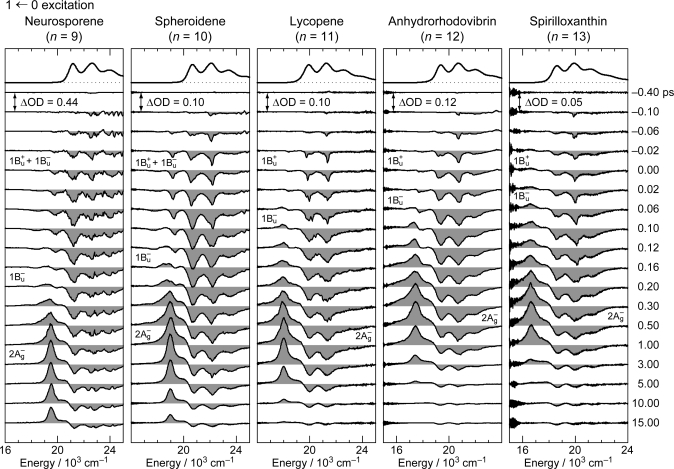
After excitation to the 1B_u_^+^(1) level, instead; see the caption of Figure 8 (Reproduced from Ref. [10]).

Figure 9 shows the pump-probe time-resolved spectra when Cars (*n* = 9–13) were excited to the 1B_u_^+^(1) level (called ‘1 ← 0 excitation’) in terms of the 1B_u_^+^ counterpart. Here, we need to remember the presence of the iso-energetic 1B_u_^−^ or 3A_g_^−^ diabatic counterpart; these diabatic pairs are shown on the top of Figure 3. We notice that the relative intensity of the strongest two stimulated-emission peaks changes with time, *not* in Car (*n* = 12) *but* in Car (*n* = 10), for example, the latter of which actually reflects vibrational relaxation (*vide infra)*.

**Figure 10.**
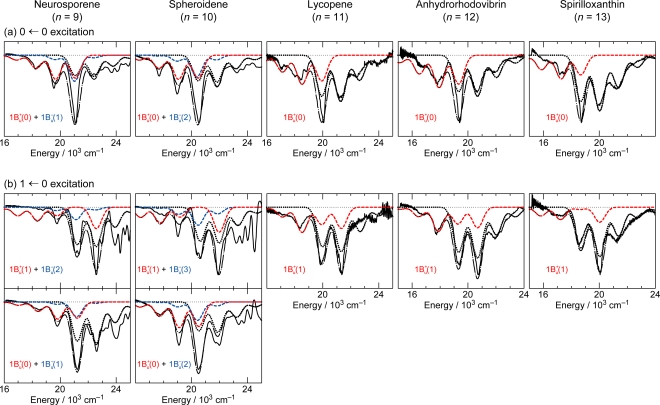
Fitting to the initial stimulated-emission profiles by the use of Franck-Condon factors for Cars (*n* = 9–13). The stimulated-emission profiles were obtained as SADS by the SVD and global-fitting analysis of data matrices, the parts of which are presented in Figure 8 and Figure 9. Specification of lines: SADS (black solid lines), stimulated emission from 1B_u_^+^ vibronic levels (red broken lines) and 1B_u_^−^ vibronic levels (blue broken lines), the bleaching of the ground-state absorption (black dotted lines) and a sum of all the contributions (black dotted-broken lines) (Reproduced from Ref. [10]).

Figure 10 exhibits the initial stimulated-emission patterns that have been extracted from the time-resolved spectra as SADS by means of the SVD and global-fitting analysis. In Cars (*n* = 9 and 10), we succeeded in time-resolving the initial two components reflecting vibrational relaxation. We tried to simulate those negative signals in terms of the following components: the 1B_u_^+^ stimulated emission (in red), the 1B_u_^−^ stimulated emission (blue) and the bleaching of ground-state absorption (black) shown in broken or dotted line. Their sums (black dotted-broken line) are compared with the observed stimulated-emission patterns as SADS (black solid line).

The results of simulation can be summarized as follows: *(a) Shorter-chain Cars (n = 9 and 10):* After the 0 ← 0 excitation, the observed stimulated emission can be explained by *simultaneous* stimulated emission from the diabatic pairs, *i.e.*, 1B_u_^+^(0) + 1B_u_^−^(1) and 1B_u_^+^(0) + 1B_u_^−^(2), respectively. After the 1 ← 0 excitation, the vibrational relaxation of 1B_u_^+^(1) + 1B_u_^−^(2) → 1B_u_^+^(0) + 1B_u_^−^(1) and 1B_u_^+^(1) + 1B_u_^−^(3) → 1B_u_^+^(0) + 1B_u_^−^(2), respectively, take place. *(b) Longer-chain Cars (n = 11–13):* After the 0 ← 0 excitation, only the 1B_u_^+^(0) stimulated emission is observed, whereas after the 1 ← 0 excitation, only the 1B_u_^+^(1) stimulated emission, instead. *Neither* the contribution of the 3A_g_^−^ counterpart *nor* the vibrational relaxation of 1B_u_^+^(1) → 1B_u_^+^(0) is observed at all in the set of SADS.

**Figure 11.**
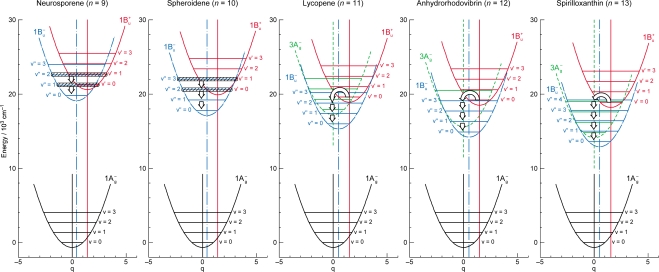
Diabatic electronic mixing between the 1B_u_^+^ and 1B_u_^−^ vibronic levels accompanying simultaneous stimulated emission in Cars (*n* = 9 and 10) and diabatic internal conversion from the 1B_u_^+^ to 1B_u_^−^ vibronic level in Cars (*n* = 11–13). In the latter Cars, neither diabatic electronic mixing nor diabatic internal conversion between the 1B_u_^+^ and 3A_g_^−^ vibronic levels takes place. Diabatically-mixed states are shadowed, and internal conversion and vibrational relaxation are shown by long bent and short straight arrows, respectively (Reproduced from Ref. [10]).

Figure 11 summarizes the relaxation dynamics starting from the diabatic pair for all the set of Cars (*n* = 9–13): The shadowed envelopes show *simultaneous* stimulated emission from the 1B_u_^+^ + 1B_u_^−^ diabatic pair, and the bent and short arrows, internal conversion and vibrational relaxation, respectively. (a) *Shorter-chain Cars (n = 9 and 10):* As revealed by the simulation described above, the simultaneous stimulated emission from the 1B_u_^+^ and 1B_u_^−^ diabatic pair and the vibrational relaxation of the diabatic pair, *i.e.*, υ = 1 → υ = 0 in terms of the 1B_u_^+^ counterpart, are seen. (b) *Longer-chain Cars (n* = 11–13*): Neither* the stimulated emission from the 3A_g_^−^ optically-forbidden counterpart *nor* the vibrational relaxation in the 1B_u_^+^ manifold is seen in Cars (*n* = 11–13). This is because the 1B_u_^+^ + 3A_g_^−^ diabatic electronic mixing never takes place, and the 1B_u_^+^ → 1B_u_^−^ diabatic internal conversion, instead, takes place very efficiently.

As shown in the pump-probe time-resolved spectra and the simulation of the initial stimulated emission, the 1B_u_^+^ + 1B_u_^−^ stimulated emission transforms into the 1B_u_^−^ transient absorption while taking vibrational relaxation in Cars (*n* = 9 and 10), whereas the 1B_u_^+^ stimulated emission directly transforms into the 1B_u_^−^ transient absorption in Cars (*n* = 11–13). Although the resultant 1B_u_^−^ transient absorption is the same, the mechanisms of its generation are different between the shorter-chain and the longer-chain Cars. It is continuous vibrational relaxation in the 1B_u_^−^ manifold in the former, while it is the 1B_u_^+^ to 1B_u_^−^ internal conversion followed by vibrational relaxation in the 1B_u_^−^ manifold in the latter (Figure 11).

In the latter case, we saw efficient 1B_u_^+^ → 1B_u_^−^ → 2A_g_^−^ internal conversion in the time-resolved spectra and in the results of SVD and global-fitting analysis of the spectral-data matrices (see Figure S3 in Supporting Information of Ref. [13]). Thus, the reason for the absence of the 3A_g_^−^ signal in the pump-probe time-resolved spectra of Cars (*n* = 11–13) (Figures 8 and 9) has been explained.

The above set of results lead us to the following symmetry selection rule, concerning the diabatic electronic mixing and diabatic internal conversion: ‘1B_u_^+^-to-1B_u_^−^ is allowed but 1B_u_^+^-to-3A_g_^−^ is forbidden’. This selection rule has been theoretically explained [10].

### 2.3. Excitation to the 1B_u_^+^(1) Level of All-Trans- and 15-*Cis-β*-Carotenes in Nonpolar and Polar Solvents [14]

**Figure 12.**
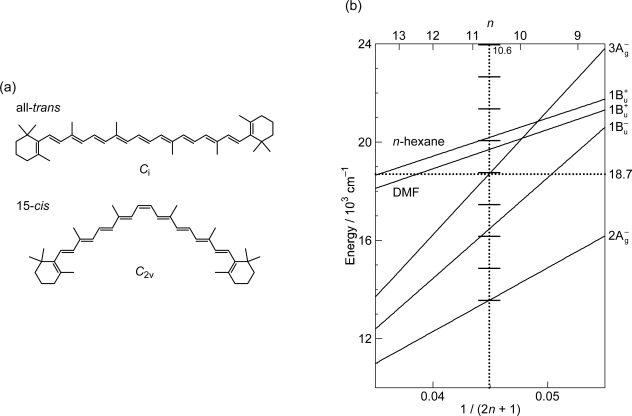
**(a)** Chemical structures of all-*trans*- and 15-*cis*-*β*-carotenes having *C*_2h_ and *C*_2v_ symmetries. **(b)** An energy diagram for the 1B_u_^+^(0) level in *n*-hexane and DMF determined by conventional electronic-absorption spectroscopy and for the 3A_g_^−^, 1B_u_^−^ and 2A_g_^−^ levels determined by measurement of resonance-Raman excitation profiles (taken from Figure 1). An effective conjugation length of *n* = 10.6 was determined by the crossing point between a horizontal line showing the 3A_g_^−^(0) energy of 18,700 cm^−^^1^ and the 3A_g_^−^ regression line shown in Figure 1. A consecutive series of vibrational ladders can be formed for the 1B_u_^+^, 3A_g_^−^, 1B_u_^−^ and 2A_g_^−^ states assuming a spacing of 1,300 cm^−^^1^ (Reproduced from Ref. [14]).

Figure 12 presents (a) the molecular structures of all-*trans*- and 15-*cis*-*β*-carotenes having *C*_i_ and *C*_2v_ symmetries and (b) an energy diagram for *β*-carotenes with the effective conjugation length of *n* = 10.6 (evaluated from the crossing point between the 3A_g_^−^(0) energy of 18,700 cm^−1^ in *β*-carotenes and the 3A_g_^−^(0) regression line reproduced from Figure 1). Here, a consecutive vibrational ladder with a spacing of 1,300 cm^−1^ including the 2A_g_^−^(0), 1B_u_^−^(0), 3A_g_^−^(0) and 1B_u_^+^(0) origins can be formed, a unique property of this particular Car.

**Figure 13.**
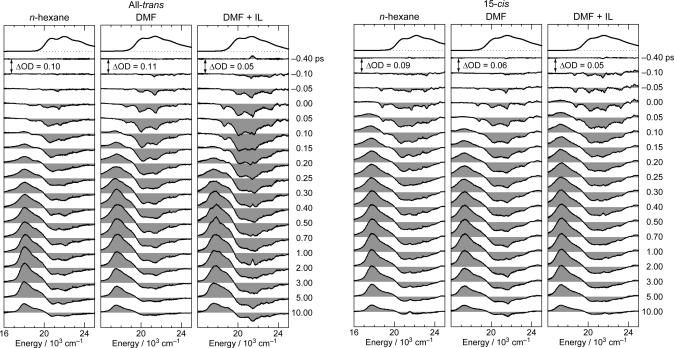
Subpicosecond time-resolved spectra after excitation with ∼100 fs pulses to the 1B_u_^+^(1) level of all-*trans*- and 15-*cis*-*β*-carotenes in *n*-hexane, DMF and DMF + IL (see text) (Reproduced from Ref. [14]).

Figure 13 shows a set of pump-probe time-resolved spectra after selective excitation with ∼100 fs pulses to the 1B_u_^+^(1) level (the absorption maximum) of all-*trans*- and 15-*cis*-*β*-carotenes in nonpolar (*n*-hexane) and polar (DMF and DMF + IL) solvents; here, DMF indicates dimethylformamide and IL, an ionic liquid, *i.e.*, methyl-3-octylimidazoluim tetrahydrofluoroborate.

**Figure 14.**
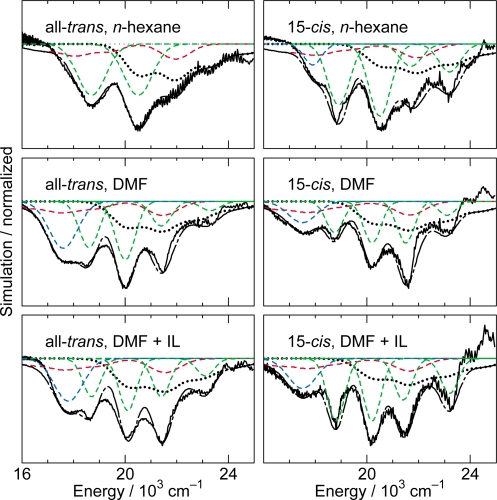
Simulation of the initial stimulated-emission patterns obtained as the first SADS by the SVD and global-fitting analysis of data matrices, parts of which are presented in Figure 13. The Franck-Condon profiles are used for the 1B_u_^+^(1) emission (red broken lines) and the bleaching of the 1B_u_^+^ ← 1A_g_^−^ absorption (black dotted lines). The Gaussian profiles are used for the 3A_g_^−^ (green broken lines) and 1B_u_^−^ (blue broken lines, as an approximation). The progression of stimulated emission peaks can be generated by resonance transfer of phonons (see text and Figure 15) (Reproduced from Ref. [14]).

Figure 14 presents the stimulated-emission patterns (black solid line) that have been obtained as the first SADS components in the SVD and global-fitting analysis of the time-resolved data matrices, parts of which are presented in Figure 13. We tried to simulate each stimulated-emission pattern by the use of the Franck-Condon profiles for the 1B_u_^+^ → 1A_g_^−^ stimulated emission (red broken line) and the bleaching of the 1B_u_^+^ ← 1A_g_^−^ absorption (black dotted line) as well as the Gaussian profiles from the 1B_u_^−^(1) (blue broken line) and 3A_g_^−^(0), 3A_g_^−^(1), 3A_g_^−^(2) and 3A_g_^−^(3) (green broken line) vibronic levels. In the calculation of the Frank-Condon simulation, we analyzed the fluorescence data of *β*-carotene [15] again to determine the 1B_u_^−^ potential treating collectively the C=C and C–C stretching modes. Here, the Gaussian profiles of the 1B_u_^−^(1) level is an approximation (actually it has a pair of wings on both sides; see Figure 10). Most importantly, the Gaussian peaks of the 3A_g_^−^(0) – 3A_g_^−^(3) levels must originate from the negligible shift of the 3A_g_^−^ potential in reference to the 1A_g_^−^ potential (to be proven in Section 3.1).

The stimulated-emission profiles of all-*trans*- and 15-*cis*-*β*-carotenes can be characterized as follows (see Figure 14): *(a) Comparison between the two isomers:* In *n*-hexane, all-*trans*-*β*-carotene exhibits a simplified stimulated-emission profile consisting of two main peaks, *i.e.*, 3A_g_^−^(0) and 3A_g_^−^(1), whereas 15-*cis*-*β*-carotene exhibits a progression consisting of five peaks, *i.e.*, 1B_u_^−^(1), 3A_g_^−^(0), 3A_g_^−^(1), 3A_g_^−^(2) and 3A_g_^−^(3), both in addition to a weak Franck-Condon profile from the 1B_u_^+^(1) level. This spectral change, on going from all-*trans*- to 15-*cis*-*β*-carotene, reflects the lowering of the symmetry of the conjugated chain from *C*_i_ to *C*_2v_. *(b) Comparison among three solvents:* In all-*trans*-*β*-carotene, the stimulated-emission peaks increase in number on going from *n*-hexane to DMF or DMF + IL. Some peaks are further enhanced in the latter polar solvent. In 15-*cis*-*β*-carotene, no changes in the number of peaks are observed on going from *n*-hexane to DMF or DMF + IL, although changes in the relative intensities are observed; 15-*cis*-*β*-carotene in DMF + IL gives rise to the clearest set of Gaussian peaks. We added IL to enhance the polarization of the conjugated chain; actually, some 3A_g_^−^ Gaussian peaks seem to be enhanced after addition of IL.

**Figure 15.**
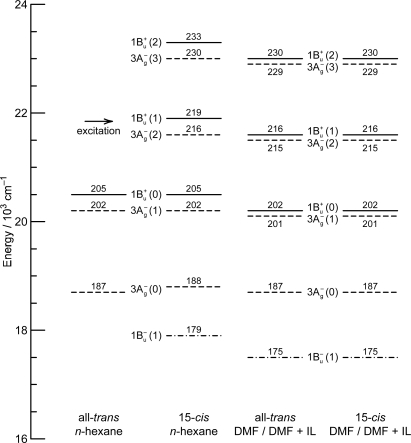
Vibronic levels of the 1B_u_^+^, 3A_g_^−^ and 1B_u_^−^ states giving rise to the stimulated emission profiles shown in Figure 14 (see text for the details) (Reproduced from Ref. [14]).

Figure 15 exhibits vibronic levels giving rise to the relevant peaks appearing as a sum of the 1B_u_^+^ → 1A_g_^−^ stimulated emission (Franck-Condon) components and the 3A_g_^−^ → 1A_g_^−^ plus 1B_u_^−^ → 1A_g_^−^ stimulated emission (Gaussian) components. The overlapped 1B_u_^+^ + 3A_g_^−^ vibronic levels as well as the isolated 3A_g_^−^(0) and 1B_u_^−^(1) levels can give rise to a progression of peaks due to the multiple 1B_u_^+^(*m*) ↔ 3A_g_^−^(*n*) or 1B_u_^−^(1) radiative resonance transitions to facilitate the mutual transfer of phonons (vibrational energy) between a pair of molecules in aggregates.

## 3. Pump-Probe Spectroscopy: Coherent Excitation

### 3.1. Excitation to the 1B_u_^+^(0) Level of Car (n = 11–13) in THF: Negligible Shift of the 3A_g_^−^ Potential [11]

We have been determining the 1B_u_^+^, 2A_g_^−^ and 1B_u_^−^ potentials of Cars by fluorescence spectroscopy: The shift of potential minimum, in reference to the ground-state 1A_g_^−^ potential, was the largest in the 2A_g_^−^ state, a middle in the 1B_u_^+^ state, and the smallest in the 1B_u_^−^ state [9], the 3A_g_^−^ potential being left to be determined. However, we saw a progression of the Gaussian-type 3A_g_^−^ stimulated emission in isomeric *β*-carotenes (see Section 2.3), each of which strongly suggested the negligible shift of the 3A_g_^−^ potential; here, the progression was ascribed to resonance transfer of phonons between a pair of Car molecules in aggregates as mentioned in Section 2.3.

**Figure 16.**
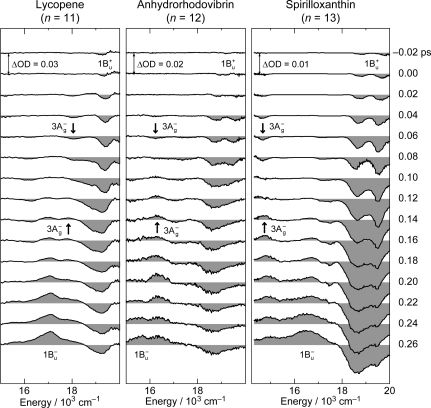
Time-resolved stimulated-emission and transient-absorption spectra after *coherent* excitation with ∼30 fs pulses aiming at the 1B_u_^+^(0) level of Cars (*n* = 11–13) in THF solution. The single 3A_g_^−^ stimulated-emission and transient-absorption peaks as well as the 1B_u_^−^ transient-absorption profile with a vibrational structure are indicated (Reproduced from Ref. [11]).

**Figure 17.**
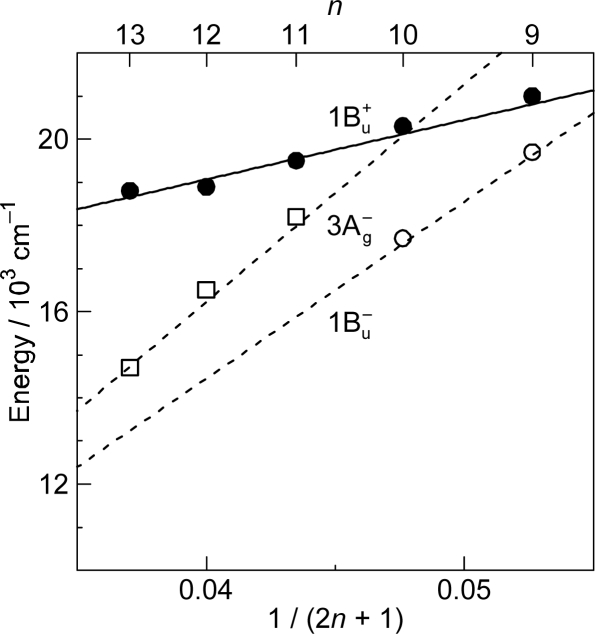
The 1B_u_^+^(0) energies of Cars (*n* = 9–13) in THF solution determined by conventional electronic-absorption spectroscopy as well as the 3A_g_^−^(0) and 1B_u_^−^(0) energies determined by the stimulated-emission peaks in Figure 16 and Figure 19, which are compared with the regression line determined by the RREP measurement of crystalline Cars (*n* = 9–13) (taken from Figure 1) (Reproduced from Ref. [16]).

Figure 16 shows a set of pump-probe time-resolved stimulated-emission and transient-absorption spectra after coherent excitation with ∼30 fs pulses to the 1B_u_^+^(0) + 3A_g_^−^ (υ = 1–3) diabatic levels of Car (*n* = 11–13) in THF solution. Following the initial 1B_u_^+^(0) stimulated emission, a set of peaks assignable to the 3A_g_^−^ *stimulated emission* appears. The stimulated emission peak systematically shifts to the lower energy with *n*, and exactly fits the regression line of the 3A_g_^−^(0) energy obtained by the measurement of resonance-Raman excitation profiles (Figure 1) as shown in Figure 17. Subsequently, a set of peaks ascribable to the 3A_g_^−^ *transient absorption* with the same energy appears as shown in the time-resolved spectra.

The time-dependent changes in the fluorescence and absorption patterns can be characterized as follows: (1) The 3A_g_^−^ stimulated emission appears first as a single peak having the 3A_g_^−^(0) energy; no vibrational structures ascribable to the Franck-Condon factors are seen at all. (2) The 3A_g_^−^ transient absorption appears next also as a single peak having the 3A_g_^−^(0) energy. No vibrational structures are seen in the 3A_g_^−^ transient absorption either. (3) The 3A_g_^−^ transient absorption is longer-lived and gets stronger in intensity than the 3A_g_^−^ stimulated emission at later delay times, the former becomes overlapped with the 1B_u_^−^ transient absorption having a vibrational structure and less clear.

**Figure 18.**
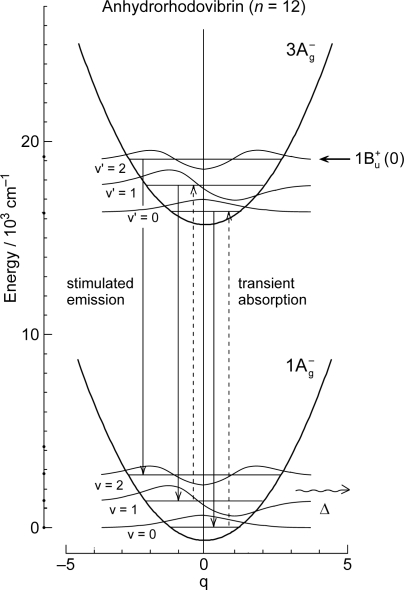
The negligible shift of the 3A_g_^−^ potential, in reference to the 1A_g_^−^ potential, a mechanism which gives rise to the transformation, with time, from a single stimulated-emission peak to a the single transient-absorption peak shown in Figure 16. Here, all the vibrational wavefunctions in the 3A_g_^−^ and 1A_g_^−^ states become orthogonal, and only the downward and upward transitions indicated by vertical arrows become allowed (Reproduced from Ref. [11]).

Figure 18 shows a mechanism proposed to explain the above time-dependent changes in the stimulated-emission and transient-absorption patterns in Car (*n* = 12), for example. The key issue here is the negligible shift of the 3A_g_^−^ potential in reference to the 1A_g_^−^ potential. Under this condition, all the vibrational wavefunctions in both the upper 3A_g_^−^ and the lower 1A_g_^−^ states become orthogonal, and *only* the iso-energetic 3A_g_^−^(2) ↔ 1A_g_^−^(2), 3A_g_^−^(1) ↔ 1A_g_^−^(1) and 3A_g_^−^(0) ↔ 1A_g_^−^(0) emissive or absorptive transitions become *allowed*. Then, all the above characteristics (i), (ii) and (iii) can be explained as follows:

(a) The Car (*n* = 12) molecules are coherently excited by absorption of photons to the 1B_u_^+^(0) + 3A_g_^−^(2) diabatic pair (see Section 3.2 for the details of this state). Following the processes of vibrational relaxation in the 3A_g_^−^ manifold, the 3A_g_^−^(2) → 1A_g_^−^(2), 3A_g_^−^(1) → 1A_g_^−^(1) and 3A_g_^−^(0) → 1A_g_^−^(0) stimulated emission, having exactly the same energy as that of the 3A_g_^−^(0) ← 1A_g_^−^(0) transition (Figure 3), should take place in this sequence. (b) A substantial amount of light energy deposited on the 3A_g_^−^(2) level should be accumulated as thermal energy on the 1A_g_^−^ vibronic levels after a while, when its dissipation is slow. This gives rise to the 3A_g_^−^(0) ← 1A_g_^−^(0), 3A_g_^−^(1) ← 1A_g_^−^(1) and 3A_g_^−^(2) ← 1A_g_^−^(2) absorptive transitions with the same transition energy as mentioned above.

Thus, the negligible shift of the 3A_g_^−^ potential, in reference to the 1A_g_^−^ potential, has been established by coherent excitation of the set of Cars (*n* = 11–13).

### 3.2. Excitation to the 1B_u_^+^(0) Level of Cars (n = 9–13) in THF: Quantum Beat Mechanism [16]

**Figure 19.**
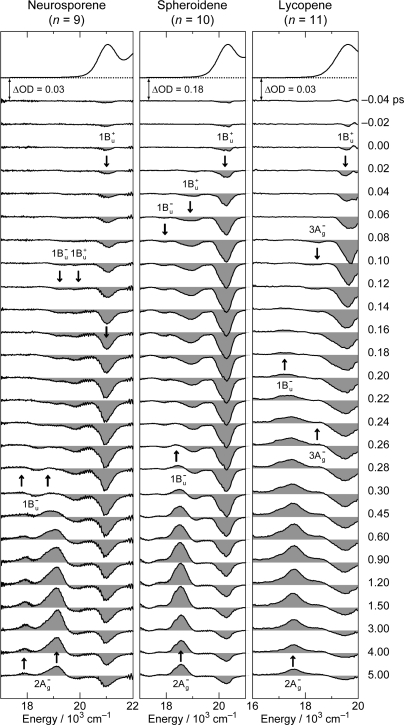
Time-resolved stimulated-emission and transient-absorption spectra after coherent excitation with ∼30 fs pulses aiming at the 1B_u_^+^(0) level of Cars (*n* = 9−11) in THF solution. After the short-lived pure 1B_u_^+^(0) emission, the strong and broad persistent peak from the 1B_u_^+^(0) + X^−^(υ) diabatic pair appears in each Car; here, the X^−^(υ) level corresponds to the 1B_u_^−^(1), 1B_u_^−^(2) and 3A_g_^−^(1) levels in Cars (*n* = 9, 10 and 11, respectively). The 1B_u_^−^(0) → 1A_g_^−^(0), 3A_g_^−^(0) → 1A_g_^−^(0) and 1B_u_^+^(0) → 1A_g_^−^(1) stimulated emission peaks are also seen together with the 1B_u_^−^ and 2A_g_^−^ transient absorption peaks (Reproduced from Ref. [16]).

The unique excited-state dynamics after coherent excitation with ∼30 fs pulses to the diabatic pair of Cars (*n* = 9–13) in THF solution stems from the following experimental conditions: (a) The interaction between the optically-allowed 1B_u_^+^(0) and the optically-forbidden iso-energetic 1B_u_^−^ or 3A_g_^−^ levels should become stronger in polar solvent than in nonpolar solvent due to the polarization of the conjugated chain and the resultant symmetry degradation from *C*_2h_ to *C*_s_. (b) The spectrally-broad ∼30 fs pulses enable the *simultaneous coherent excitation* of the diabatic pair as mentioned above. (c) The photon density (the number of photons per unit time) should be much higher in ∼30 fs pulses than in ∼100 fs pulses; therefore, there is a good chance that the diabatic pair becomes densely populated. These conditions gave rise to unique spectral patterns (Figure 19) that are *completely different* from those obtained by selective excitation with ∼100 fs pulses in nonpolar solvent (Figure 8). Here, the Franck-Condon simulation of the stimulated emission is impossible.

**Figure 20.**
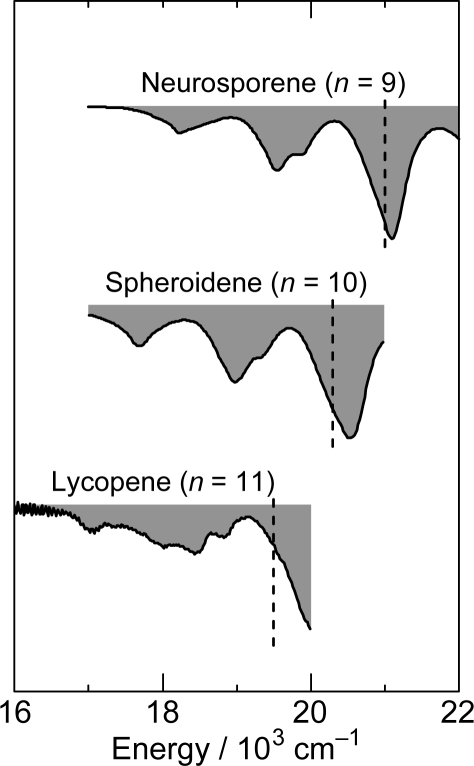
A set of stimulated-emission patterns obtained by *selective excitation* with ∼100 fs pulses to the 1B_u_^+^(0) level of Cars (*n* = 9–11) in nonpolar solvent (taken from Figure 10). They are to be compared with the stimulated-emission patterns obtained by *coherent excitation* with ∼30 fs pulses to the 1B_u_^+^(0) level of the same set of Cars in THF solution (Figure 19). The same spectral region is presented to facilitate comparison.

To facilitate the spectral comparison, the initial stimulated-emission patterns that have been presented in Figure 8 are reproduced for Cars (*n* = 9–11) in Figure 20, in the spectral regions corresponding to those of Figure 19 are presented. The vertical broken lines indicate the positions of the predominant stimulated-emission peaks in Figure 19 (note the difference in solvent polarity between *n*-hexane and THF).

The pump-probe time-resolved spectra of Cars (*n* = 9–11) presented in Figure 19, that have been recorded under the above-mentioned experimental conditions (a), (b) and (c), can be characterized as follows: *(i) An initial short-lived stimulated-emission peak from the 1B_u_^+^(0) level.* Immediately after excitation, a weak and sharp peak ascribable to the pure 1B_u_^+^(0) stimulated emission appears. *(ii) A persistent stimulated emission peak from the 1B_u_^+^(0) + X^−^(υ) diabatic pair*. (Here, X^−^(υ) indicates the 1B_u_^−^(1), 1B_u_^−^(2), 3A_g_^−^(1), 3A_g_^−^(2) and 3A_g_^−^(3) levels for Cars (*n* = 9, 10, 11, 12 and 13, respectively).) Subsequently, a much stronger, broad and long-lived stimulated-emission peak ascribable to the 1B_u_^+^ + X^−^(υ) diabatic pair appears. This particular peak even tends to split into two in Car (*n* = 10) in the 0.15–0.30 ps time region. Eventually, the stimulated emission from the diabatic pair disappears and the bleaching of the ground-state 1B_u_^+^(0) ← 1A_g_^−^(0) absorption and the 2A_g_^−^ transient-absorption remain. *(iii) The 1B_u_^−^(0)*, *3A_g_^−^(0) and 1B_u_^+^(0) stimulated emission peaks.* Around 0.06–0.10 ps, a peak systematically shifting to the lower energy with *n*, which is ascribable to the 1B_u_^−^(0) stimulated emission, appears in Cars (*n* = 9 and 10). As shown in Figure 17, the energies of these stimulated emission peaks agree with those determined by RREP measurement. In the shorter-chain Cars (*n* = 9 and 10), another 1B_u_^+^(0) stimulated emission ascribable to the 1B_u_^+^(0) → 1A_g_^−^(1) transition appears, while stimulated emission from the 1B_u_^+^(0) + X^−^(υ) diabatic pair predominates. Later, both the 1B_u_^+^(0) and 1B_u_^−^(0) stimulated emission peaks are replaced by the 1B_u_^−^ transient absorption peaks.

**Figure 21.**
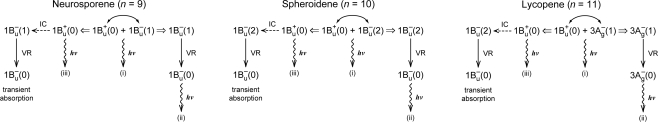
Schemes of relaxation processes when the set of Cars (*n* = 9–11) is coherently excited with ∼30 fs pulses to the 1B_u_^+^(0) + X^−^(υ) diabatic pairs (for the notation of X^−^(υ), see the caption of Figure 19): The initial set of stimulated emissions can be explained in terms of quantum beat. In Car (*n* = 10), for example, it consists of (i) the persistent stimulated emission from the 1B_u_^+^(0) + 1B_u_^−^(2) diabatic pair as the coherent cross term, (ii) the short-lived 1B_u_^+^(0) stimulated emission as one of the split incoherent terms and (iii) the short-lived 1B_u_^−^(0) stimulated emission as the other split incoherent term. Also, the 1B_u_^+^(0) species internally-converts to 1B_u_^−^(2) and, then, vibrationally-relaxes to the 1B_u_^−^(0) species, which gives rise to transient absorption. In Car (*n* = 11), 1B_u_^−^(2) is replaced by 3A_g_^−^(1) in (i) and (iii).

The above spectral characteristics can be explained in terms of the mechanism of quantum beat (see Figure 21 referring to Figure 19). To be consistent to what will be described in Section 3.3, we will take the case of Car (*n* = 10) as the first example: (i) The stimulated emission from the 1B_u_^+^(0) + 1B_u_^−^(2) diabatic pair, which follows the initial weak stimulated emission from the pure 1B_u_^+^(0) level, can be attributed to the coherent cross term. In phenomenological expression, the 1B_u_^+^(0) lifetime becomes *substantially* lengthened by back-and-forth exchanges between the optically-allowed 1B_u_^+^ and optically-forbidden 1B_u_^−^ states; for the rigorous theoretical description of this quantum beat mechanism, see Eq. 48 in Ref. [10]. (ii) The 1B_u_^−^(0) level that is responsible for stimulated emission is the result of vibrational relaxation from the 1B_u_^−^(2) optically-forbidden counterpart of the diabatic pair; this is attributed to one of the incoherent terms. (iii) The 1B_u_^+^(0) level exhibits strong 1B_u_^+^(0) → 1A_g_^−^(1) stimulated emission in this particular Car; this is ascribable to the other incoherent term.

Next, we proceed to the case of Car (*n* = 11), which can be explained in a similar way, where 1B_u_^−^(2) is replaced by 3A_g_^−^(1) as shown in Figure 21: (i) The stimulated emission from the 1B_u_^+^(0) + 3A_g_^−^(1) pair (which follows the initial weak stimulated emission from the pure 1B_u_^+^(0) level) exhibits a strong, broad and long-lived peak. (ii) The 3A_g_^−^(0) stimulated emission is weakly observed. (iii) The pure 1B_u_^+^(0) → 1A_g_^−^(0) stimulated emission is probably hidden in the diabatic 1B_u_^+^(0) + 3A_g_^−^(1) profile. The stimulated emission due to the 1B_u_^+^(0) → 1A_g_^−^(1) transition is not clearly seen at all, most probably it is overlapped with the 3A_g_^−^(0) → 1A_g_^−^(0) stimulated emission.

Thus, in terms of the quantum beat formalism, the 1B_u_^+^(0) + X^−^(υ) stimulated emission is ascribable to the coherent cross term, while the 1B_u_^+^(0) and the X^−^(0) stimulated emission, to the pair of split incoherent terms.

**Figure 22.**
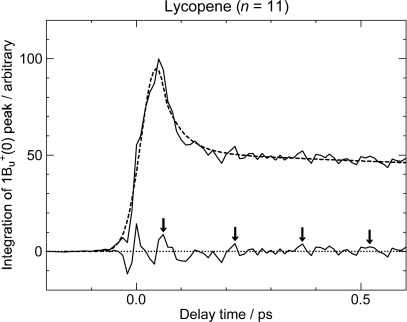
Time-dependent changes in the integrated intensity of stimulated emission at the position of the 1B_u_^+^(0) energy in Car (*n* = 11). The initial decay phase reflects the pure 1B_u_^+^(0) stimulated emission and the later stationary phase, stimulated emission from the 1B_u_^+^(0) + 3A_g_^−^(1) diabatic pair. After the subtraction of the background time profile, an oscillatory change emerges. The result supports the presence of the persistent stimulated emission generated by the quantum beat mechanism (Reproduced from Ref. [16]).

Finally, the key question is whether we can actually observe the real quantum beat: Figure 3 shows that the energy gap between the 1B_u_^+^(0) and 3A_g_^−^(1) pair of Car (*n* = 11) is around 300 cm^−1^, whereas that between the 1B_u_^+^(0) and 1B_u_^−^(2) levels of Car (*n* = 10) is almost zero. Therefore, there is a good chance of observing the real quantum beat *not* in Car (*n* = 10) *but* in Car (*n* = 11). Figure 22 shows the time-dependent changes in the integrated intensity of stimulated emission from the 1B_u_^+^(0) + 3A_g_^−^(1) diabatic pair in Car (*n* = 11). After the subtraction of the background time profile, we can actually see the oscillatory changes in the fluorescence intensity. This result strongly supports our explanation of the observed spectral characteristics in terms of the quantum-beat mechanism.

### 3.3. Excitation to the 1B_u_^+^(0) Level of Cars (n = 9–11) Bound to LH2 Antenna Complexes [17]

As shown in Section 3.2, the coherent excitation of Cars (*n* = 9–13) in a polar solvent, THF, exhibited *not only* the long-lived stimulated emission from the 1B_u_^+^(0) + X^−^(υ) diabatic pair *but also* the short-lived stimulated emission from the split 1B_u_^+^(0) and X^−^(0) levels, which have been explained in terms of the quantum-beat mechanism. Therefore, we have tried to find whether the excited-state dynamics, after the coherent excitation of Cars (*n* = 9–11) that are bound to LH2 antenna complexes, is similar to, or different from, the excited-state dynamics free in THF solution.

**Figure 23.**
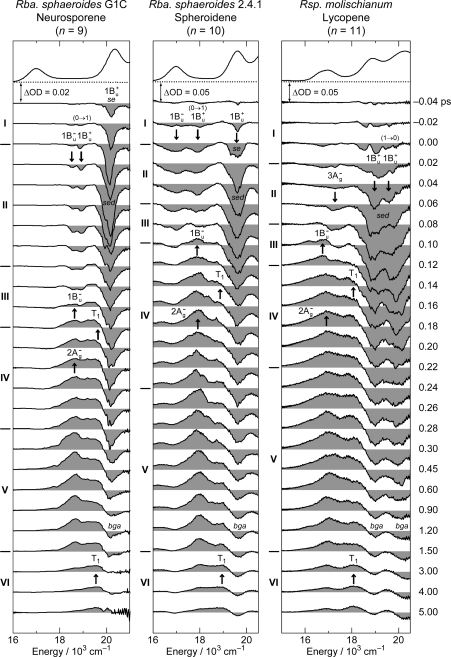
Time-resolved stimulated-emission and transient-absorption spectra after coherent excitation with ∼30 fs pulses aiming at the 1B_u_^+^(0) levels of Cars (*n* = 9–11) bound to LH2 antenna complexes from *Rba. sphaeroides* G1C, *Rba. sphaeroides* 2.4.1.and *Rsp. molischianum*. The origins of the relevant electronic states, in stimulated emission and transient absorption, are indicated together with specific vibronic transitions when necessary; ‘*se*’, ‘*sed*’ and ‘*bga*’ labeled on peaks indicate pure stimulated emission, stimulated emission from the diabatic pair, and bleaching of the ground-state absorption, respectively (Reproduced from Ref. [17]).

*Excited-state dynamics as revealed by time-resolved spectra and by the results of SVD and global-fitting analysis.* Figure 23 shows the pump-probe time-resolved stimulated-emission and transient-absorption spectra of Cars (*n* = 9–11) bound to LH2 complexes from *Rba. sphaeroides* G1C, *Rba. sphaeroides* 2.4.1 and *Rsp. molischianum*. The time-resolved spectra can be characterized as follows:

*(a) Cars (n = 9 and 10).* Following the weak 1B_u_^+^(0) stimulated emission, strong stimulated emission from the 1B_u_^+^(0) + X^−^(υ) diabatic pair appears, giving rise to a broad peak or even clearly-split two peaks. Simultaneously, the 1B_u_^+^(0) → 1A_g_^−^(1) and the 1B_u_^−^(0) → 1A_g_^−^(0) weak stimulated emission peaks emerge. The set of stimulated emission components can be explained in terms of the quantum beat mechanism, as in the case of these Cars in THF solution. The latter pair of stimulated emission is replaced by the 1B_u_^−^ transient absorption, and then by the combination of the *m*B_u_^+^ ← 2A_g_^−^ and T_n_ ← T_1_ transient absorption peaks.

**Figure 24.**
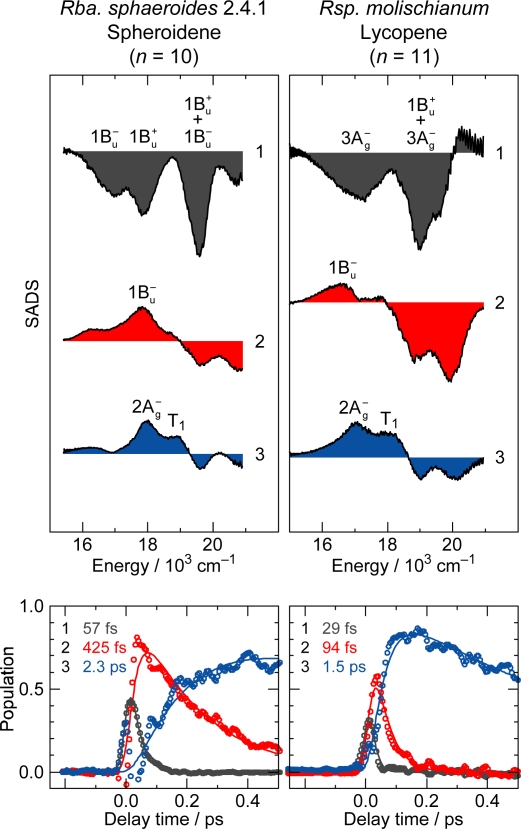
The results of SVD followed by global fitting of time-resolved data matrices of Cars (*n* = 10 and 11) bound to the LH2 complexes from *Rba. sphaeroides* 2.4.1 and *Rsp. molischianum*, respectively, after coherent excitation with ∼30 fs pulses aiming at the 1B_u_^+^(0) level. A set of SADS (upper panels) and time-dependent changes in population (lower panels) are shown. The sequential changes in the stimulated-emission and transient-absorption patterns (SADS) extracted from time-resolved spectra (Figure 23) and their time-dependent changes in population lead to schemes shown in Figure 25 (Reproduced from Ref. [17]).

**Figure 25.**
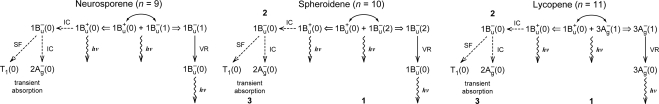
Relaxation schemes after coherent excitation to the 1B_u_^+^(0) level of Cars (*n* = 9–11) bound to the LH2 complexes: The initial set of stimulated emission based on the quantum-beat mechanism is followed by transient absorptions: ‘VR’, ‘IC’ and ‘SF’ stand for vibrational relaxation, internal conversion and singlet fission. The numbering in the schemes corresponds to that of SADS shown in Figure 24; see also Figure 21 for comparison (Reproduced from Ref. [17]).

The above sequence of events can be proven by the SVD and global-fitting analysis of bound Car (*n* = 10), for example; the SADS and time-dependent changes in population are presented in Figure 24 (left-hand-side). The sequential transformation of Component 1 → Component 2 → Component 3 is ascribable to spectral transformation, *i.e.*, a set of the 1B_u_^+^ + 1B_u_^−^, 1B_u_^+^ and 1B_u_^−^ stimulated emission → the 1B_u_^−^ transient absorption → the 2A_g_^−^ and T_1_ transient absorption. The transformation is schematically presented in Figure 25 (in the middle).

*(b) Car (n = 11).* As shown in Figure 23, after the initial 1B_u_^+^ emission (whose structure is not clear), the strong and extremely-broad pair of stimulated-emission signals ascribable to the 1B_u_^+^(0) + 3A_g_^−^(1) and 1B_u_^+^(1) + 3A_g_^−^(2) diabatic pairs appear. Simultaneously, a weak stimulated emission signal ascribable to the 3A_g_^−^(0) → 1A_g_^−^(0) transition appears and becomes replaced by the positive 1B_u_^−^ transient-absorption signal. Eventually, it transforms into the 2A_g_^−^ and T_1_ transient-absorption signals.

The above sequence of events has been proven by the SVD and global-fitting analysis of the spectral data matrix, the results of which are shown in Figure 24 (right-hand-side): The sequential transformation of Component 1 → Component 2 → Component 3 is ascribable to spectral transformation *i.e.*, the pair of the 1B_u_^+^ + 3A_g_^−^ and 3A_g_^−^ stimulated emission → the 1B_u_^−^ transient absorption → the 2A_g_^−^ and T_1_ transient absorption. The transformation is schematically presented in Figure 25 (on the right end).

**Figure 26.**
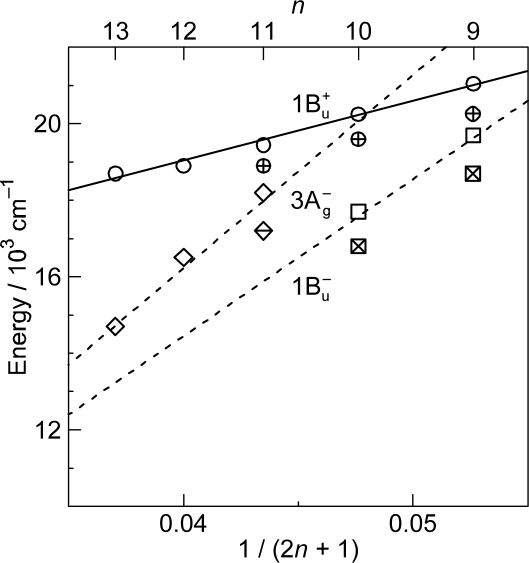
Comparison of the 1B_u_^+^(0), 1B_u_^−^(0) and 3A_g_^−^(0) energies of Cars (*n* = 9–11) bound to LH2 antenna complexes (crossed symbols) to those free in THF solution (open symbols). The 1B_u_^+^(0) energies were determined by conventional absorption spectroscopy, whereas the 1B_u_^−^(0) and 3A_g_^−^(0) energies, by the stimulated emission from these states obtained by coherent excitation of the diabatic pair, 1B_u_^+^(0) + X^−^(υ) (Reproduced from Ref. [17]).

*The singlet-state energies of Cars (n = 9–11) when bound to the LH2 antenna complexes.* Figure 26 compares the 1B_u_^+^(0), 1B_u_^−^(0) and 3A_g_^−^(0) energies of Cars (*n* = 9–11) bound to the LH2 antenna complexes (crossed symbols) to those free in THF solution (open symbols). Surprisingly, the low-energy shift upon binding of the Cars is even larger in the covalent 1B_u_^−^(0) and 3A_g_^−^(0) levels than in the ionic 1B_u_^+^(0) level. The results strongly suggest the polarization of the conjugated chain, which must enhance the electronic mixing of the diabatic pair.

*Unique excited-state dynamics of Cars bound to LH2 complexes.* The excited-state dynamics of the bound Cars (*n* = 9–11) can be characterized as follows: (a) The substantially-higher intensity of stimulated emission from the optically-forbidden 1B_u_^−^(0) and 3A_g_^−^(0) levels as well as the much broader stimulated emission from the 1B_u_^+^(0) + X^−^(υ) diabatic pair strongly support the idea of enhanced polarization of the Car conjugated chain and the resultant stronger diabatic interaction. (b) The much faster decay of the initial stimulated emission after the coherent excitation of the diabatic pair obviously reflects the branching pathway to the Car-to-BChl singlet-energy transfer in addition to internal conversion within Car. This pathway of Car-to-BChl singlet-energy transfer has been established as introduced in Section 1.1. (c) The most conspicuous change, upon the binding of Cars, is the efficient triplet generation. We have already shown that the triplet generation is due to singlet heterofission from the 1B_u_^−^ state [1]. The high efficiency of triplet generation suggests a substantial twisting around the C=C bonds in the Car conjugated chain when bound to the apo-peptides and BChls.

## 4. Kerr-Gate Fluorescence Spectroscopy

### Excitation to the 1B_u_^+^(υ = 3 or 4) Vibronic Level of Cars (n = 9–12) in Nonpolar Solvent and Probing the 1B_u_^+^(2) → 1B_u_^+^(1) → 1B_u_^+^(0) Vibrational Relaxation [9]

Our preliminary Kerr-gate fluorescence spectroscopy was found contradictory in our series of attempts to determine the 1B_u_^−^ lifetime of neurosporene, Car (*n* = 9), for example: As described in Section 2.1, we obtained a value of 240 fs (Table 1) and a similar value of 265 fs [18] by excitation of this Car to the 1B_u_^+^(0) level and probing by the use of the NIR white continuum. By subpicosecond time-resolved Raman spectroscopy, we obtained a value of 250 fs [19], whereas by fluorescence upconversion spectroscopy, a value of 270 fs, as the 1B_u_^−^ lifetime after excitation to the 1B_u_^+^(0) level [20]. Thus, the 1B_u_^−^ lifetime has been consistently determined to be in the range of 240–270 fs.

In the previous Kerr-gate fluorescence spectroscopy, after excitation to the 1B_u_^+^ (3) level, however, we obtained the 1B_u_^+^(0) lifetime of 260 fs, the value of which agreed with the 1B_u_^−^ lifetime [21]. This result confused us. Therefore, we have decided to examine a set of Cars (*n* = 9–12) after excitation at 12,500 cm^−1^, which is just below and above the 1B_u_^+^(3) level in Car (*n* = 9 and 10) and around the 1B_u_^+^(4) level in Cars (*n* = 11 and 12). The results actually provided us with deeper insight into the unique excited-state properties of the diabatic pairs, which has turned out to be the reason for the *apparent contradiction*.

**Figure 27.**
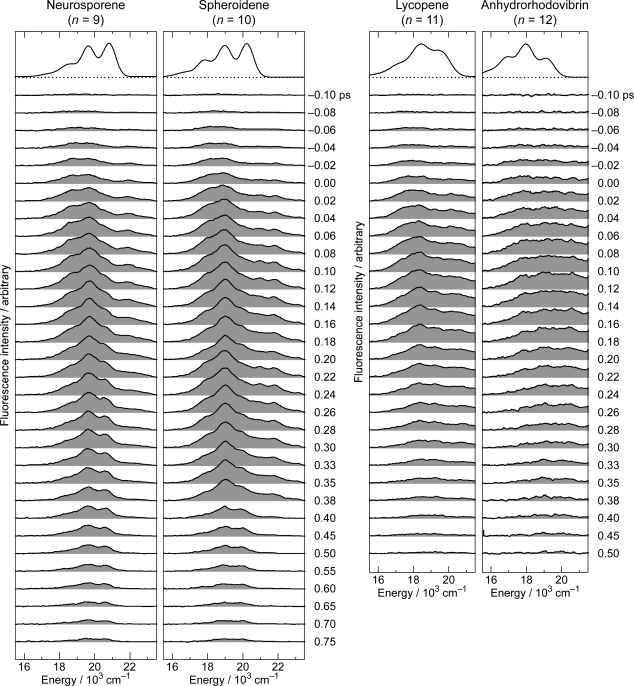
Time-resolved Kerr-gate fluorescence spectra after selective excitation with ∼100 fs pulses to the 1B_u_^+^(3) level of the shorter-chain Cars (*n* = 9 and 10) and to the 1B_u_^+^(4) level of the longer-chain Cars (*n* = 11 and 12) (Reproduced from Ref. [9]).

Figure 27 shows the time-resolved fluorescence spectra of Cars (*n* = 9–12). The spectra of the longer-chain Cars (*n* = 11 and 12) can be contrasted to those of the shorter-chain Cars (*n* = 9 and 10) as follows: (a) Fluorescence decays faster in the longer-chain Cars than in the shorter-chain Cars. (b) The spectral profile is more stretched and the relative intensity in the higher-energy region is enhanced in the longer-chain Cars than in the shorter-chain Cars. (c) In each Car, the fluorescence maximum shifts to the higher energies with time, but this trend is less pronounced in the longer-chain Cars than in the shorter-chain Cars. The time-dependent high-energy shift is ascribable to the Franck-Condon factors and reflects the vibrational relaxation in the 1B_u_^+^ manifold (*vide infra*). Since the rate of vibrational relaxation from υ = ℓ to υ = ℓ–1 is proportional to the quantum number of the starting vibrational level, ℓ [22], hopefully, we would be able to time-resolve the last two steps of the slowest vibrational relaxations.

**Figure 28.**
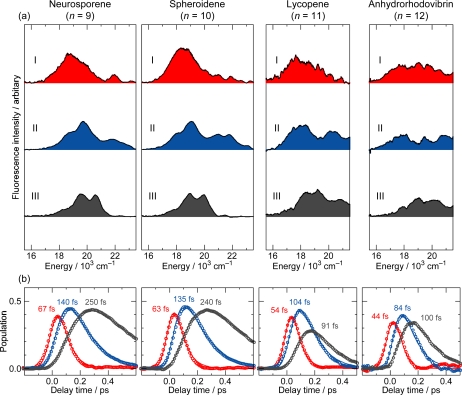
Results of SVD followed by global fitting of fluorescence data matrices of Cars (*n* = 9–12) by the use of a 3-component sequential model, including species-associated fluorescence spectra (SAFS) (upper panels) and time-dependent changes in population with decay time constants (lower panels) (Reproduced from Ref. [9]).

Figure 28 shows the results of the SVD and global-fitting analysis of spectral-data matrices by the use of a 3-component sequential model: The species-associated fluorescence spectra (SAFS) with reasonable S/N ratios (shown in the upper panels) nicely reflect the time-dependent changes in the fluorescence spectra (Figure 27). The decay time constants indicated on the time-dependent changes in population for the I, II and III components (shown in the lower panels) can be characterized as follows: (a) The lifetimes of components I and II are approximately in the ratio of 1:2, supporting their assignment to the 1B_u_^+^(2) and 1B_u_^+^(1) starting levels of vibrational relaxation in terms of the optically-allowed 1B_u_^+^ counterpart. (b) The lifetimes of component III are 250 and 240 fs in Cars (*n* = 9 and 10), whereas 91 and 100 fs in Cars (*n* = 11 and 12); the values approximately agree with the 1B_u_^−^ and 3A_g_^−^ lifetimes of the corresponding the shorter-chain and the longer-chain Cars listed in Table 1. In the comparison of those values, we need to take into account the fact that the present result is just on the edge of our spectroscopic and analytical techniques.

**Figure 29.**
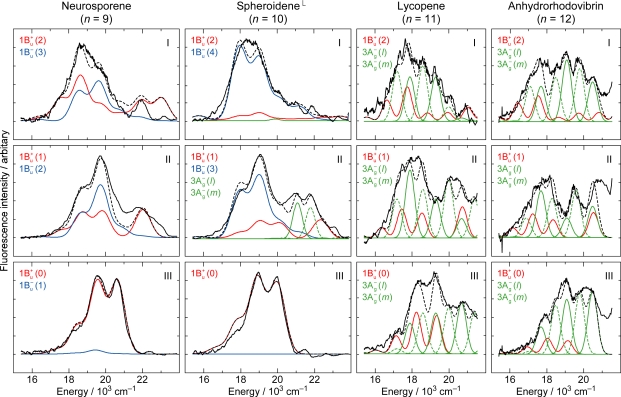
Simulation of fluorescence patterns obtained as SAFS (taken from Figure 28) by the use of the Franck Condon-type downward transitions from the 1B_u_^+^ and 1B_u_^−^ vibronic levels and a pair of progressions of the Gaussian-type downward transitions from the 3A_g_^−^ vibronic levels (Reproduced from Ref. [9]).

Figure 29 shows the results of simulation for the fluorescence patterns obtained as a set of SAFS, which can be characterized as follows: (a) In Cars (*n* = 9 and 10), fluorescence patterns I and II can be simulated in terms of the Franck-Condon factors for the downward transitions from each of the 1B_u_^+^ and 1B_u_^−^ diabatic pairs (shown in red and blue lines), but fluorescence pattern III is dominated by transition from the 1B_u_^+^(0) level. In the fluorescence pattern II of Car (*n* = 10), the contribution of the 3A_g_^−^ transitions is also seen as expected from the energy diagram (Figure 1). (b) In Cars (*n* = 11 and 12), the broad fluorescence profiles, I, II and III, could be simulated by the Franck-Condon factors for transitions from the 1B_u_^+^(2), 1B_u_^+^(1) and 1B_u_^+^(0) counterparts (shown in red) and by a pair of the 3A_g_^−^ progressions designated as 3A_g_^−^(*l*) and 3A_g_^−^(*m*) (shown in green). As described in Section 2.3, we saw a single 3A_g_^−^ progression in all-*trans*-*β*-carotene in polar solvent as well as 15-*cis*-*β*-carotene in polar and nonpolar solvents (Figure 14). Here, we temporarily ascribe the pair of progressions to the crystalfield splitting due to the intermolecular interaction of a pair of Car molecules in aggregates; note that we used a concentration as high as ∼10^−4^ M (facilitating aggregate formation) to record very weak fluorescence. Here, the contribution of the 3A_g_^−^ fluorescence predominates not only in fluorescence profiles I and II, but also in fluorescence profile III.

Thus, the fluorescence patterns can be clearly classified into two groups: one, in the shorter-chain Cars (*n* = 9 and 10) and the other, in the longer-chain Cars (*n* = 11 and 12). Most importantly, we have obtained *not only* the 1B_u_^+^-to-1B_u_^−^ diabatic electronic mixing in the shorter-chain Cars *but also* the 1B_u_^+^-to-3A_g_^−^ diabatic electronic mixing in the longer-chain Cars (11 and 12). The results indicate that symmetry degradation takes place while the Car molecules are being *vibronically excited*, presumably due to the twisting of the conjugated chain around the C=C bond(s). Then, the symmetry selection rule concerning the diabatic electronic mixing (see Section 2.2) breaks down, and the 1B_u_^+^-to-3A_g_^−^ diabatic electronic mixing becomes allowed.

**Figure 30.**
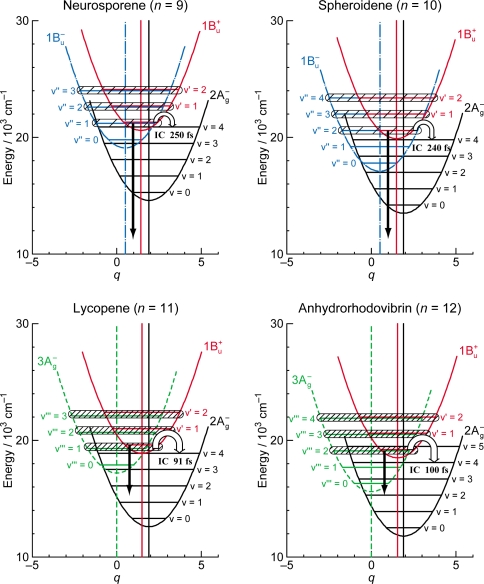
Mechanisms why the apparent 1B_u_^+^(0) lifetime agrees with the 1B_u_^−^ lifetime in Cars (*n* = 9 and 10) or with the 3A_g_^−^ lifetime in Cars (*n* = 11 and 12). After vibrational relaxation to the bottom of the 1B_u_^+^ potential, the 1B_u_^+^(0) diabatic counterpart relaxes through stimulated emission, whereas the 1B_u_^−^ (3A_g_^−^) diabatic counterpart in the former (in the latter) set of Cars through internal conversion to the iso-energetic 2A_g_^−^ vibronic level with the 1B_u_^−^ (3A_g_^−^) lifetime (Reproduced from Ref. [9]).

Now, we will try to explain why the lifetime of ‘the apparent 1B_u_^+^(0) level’ agrees with the 1B_u_^−^ lifetime in Cars (*n* = 9 and 10), whereas with the 3A_g_^−^ lifetime in Cars (*n* = 11 and 12): Figure 30 presents the mechanisms: Here, each of the mixed diabatic pair is shown by a shadowed envelope, where the density of inclined lines is set proportional to the contribution of the optically-forbidden 1B_u_^−^ or 3A_g_^−^ counterpart. In considering the relaxation processes, we need to consider the selection rule in relation to the Pariser’s ± labels (see Section 1.2).

(a) In the shorter-chain Car (*n* = 9), for example, the vibrational relaxation, 1B_u_^+^(2) + 1B_u_^−^(3) → 1B_u_^+^(1) + 1B_u_^−^(2) → 1B_u_^+^(0) + 1B_u_^−^(1), takes place first. When the diabatic pair has reached to the bottom of the 1B_u_^+^(0) potential, the allowed relaxation for the 1B_u_^+^(0) counterpart is the instantaneous 1B_u_^+^(0) emission, whereas the allowed relaxation for the 1B_u_^−^(1) counterpart is the 1B_u_^−^(1) → 2A_g_^−^(4) internal conversion. Therefore, it is quite natural that the time constant of the latter process corresponds to the 1B_u_^−^ lifetime. Actually, the allowed 1B_u_^−^-to-2A_g_^−^ internal conversion is taking place *so* efficiently from the upper 1B_u_^−^ vibronic levels *that* the 1B_u_^−^ population has been almost exhausted before the diabatic pair reaches to the bottom of the 1B_u_^+^(0) potential.

(b) In the longer-chain Car (*n* = 11), for example, after the vibrational relaxation in the sequence of 1B_u_^+^(2) + 3A_g_^−^(3) → 1B_u_^+^(1) + 3A_g_^−^(2) → 1B_u_^+^(0) + 3A_g_^−^(1), the 1B_u_^+^(0) stimulated emission and the 3A_g_^−^(1) → 2A_g_^−^(4) internal conversion are to take place; the former must take place instantaneously, while the latter, with the 3A_g_^−^ lifetime. Here, the 3A_g_^−^(1) level is still highly populated when the diabatic pair reaches to the bottom of the 1B_u_^+^ potential.

This pair of observations reflects the unique excited-state properties of the diabatic pair consisting of the optically-allowed 1B_u_^+^ counterpart and the optically-forbidden 1B_u_^−^ or 3A_g_^−^ counterpart. When the diabatic pair relaxes down to the bottom of the 1B_u_^+^ potential, the 1B_u_^+^ counterpart relaxes through emission, whereas the 1B_u_^−^ or 3A_g_^−^ counterpart relaxes through internal conversion. This is actually the splitting processes of the diabatic pair. The relative contribution of the optically-allowed and the optically-forbidden counterparts seems to vary in the processes of vibrational relaxation.

## 5. Summary and Future Trend

Pump-probe stimulated-emission and transient-absorption spectroscopy *after selective excitation* with ∼100 fs pulses to the 1B_u_^+^(0) and 1B_u_^+^(1) levels of Cars (*n* = 9–13) *in nonpolar solvent* showed the symmetry selection rule of diabatic electronic mixing and diabatic internal conversion, *i.e*., ‘1B_u_^+^-to-1B_u_^−^ is allowed but 1B_u_^+^-to-3A_g_^−^ is forbidden’. On the other hand, Kerr-gate fluorescence spectroscopy after selective excitation to the 1B_u_^+^(3) or 1B_u_^+^(4) level of Cars (*n* = 9–12) in nonpolar solvent showed the breakdown of the symmetry selection rule in the 1B_u_^+^(2) → 1B_u_^+^(1) → 1B_u_^+^(0) vibrational relaxation processes, *i.e.*, the 1B_u_^+^-to-3A_g_^−^ diabatic electronic mixing and diabatic internal conversion become allowed. The results indicate that the symmetry selection rule holds immediately after excitation, but it breaks down while the Car molecules are being excited probably due to the degradation of the *C*_2h_ symmetry of the conjugated chain.

The above results demonstrate that *not only* the 1B_u_^−^ state *but also* the 3A_g_^−^ state can play important roles in the light-harvesting function: While the Cars (*n* = 11–13) molecules are being excited they can efficiently transfer ‘the 3A_g_^−^ energy’ to BChl as far as the pair of pigment molecules are in close contact.

Pump-probe stimulated-emission and transient-absorption spectroscopy *after coherent excitation* with ∼30 fs pulses to the 1B_u_^+^(0) level of Cars (*n* = 9–13) *in polar solvent* gave rise to three stimulated-emission components, explained by the quantum beat mechanism, including the long-lived coherent cross term from the 1B_u_^+^ + 1B_u_^−^ or 1B_u_^+^ + 3A_g_^−^ diabatic pair and the short-lived 1B_u_^+^ and 1B_u_^−^ or 3A_g_^−^ split incoherent terms. The lifetimes of the coherent terms from the diabatic pairs reach as long as ∼2.5 × 10^2^ fs. Basically the same type of stimulated-emission components were identified in Cars (*n* = 9–11) bound to LH2 complexes. Actually, the substantial shortening of the stimulated emission components were observed, supporting the idea of the Car-to-BChl singlet-energy transfer.

The results strongly suggest that the coherent excitation of the diabatic pairs strongly facilitate the light-harvesting function. A key question here is whether such coherent excitation of Cars can take place *in Nature*. Preliminary four-wave-mixing (FWM) spectroscopy of Car (*n* = 10) showed coherence transfer from the 1A_g_^−^(0) → 1B_u_^+^(0) to the 1A_g_^−^(0) → 3A_g_^−^(0) transition as well as from the 1A_g_^−^(0) → 1B_u_^−^(1) to the 1A_g_^−^(0) → 1B_u_^+^(0) transition. Further, FWM spectroscopy of Car (*n* = 11) showed coherence coupling between the 1A_g_^−^(0) → 1B_u_^+^(0) and the 1A_g_^−^(0) → 3A_g_^−^(1) transitions, which decayed with a coherence lifetime of as long as 1.06 ps.

Therefore, there is a good chance that all the transitions eventually become coherently-coupled with one another and share the same phase while a set of Car molecules are being excited. (This reminds us the case where a pair of pendular hanging on the both ends of a bar eventually becomes synchronized.) Obviously, *coherence dynamics* beyond *population dynamics* is the key issue in the future in studying the dynamics of Car singlet-excited states and Car-to-BChl singlet-energy transfer.

## 6. Conclusions

Pump-probe spectroscopy after *selective excitation* of all-*trans* Cars (*n* = 9–13) in nonpolar solvent, probing stimulated emission and transient absorption, identified a symmetry selection rule of diabatic electronic mixing and diabatic internal conversion, *i.e.,* ‘1B_u_^+^-to-1B_u_^−^ is allowed but 1B_u_^+^-to-3A_g_^−^ is forbidden’. Kerr-gate fluorescence spectroscopy showed that this selection rule breaks down, due to the symmetry degradations when the Car molecules are being excited, and, as a result, the 1B_u_^+^-to-3A_g_^−^ diabatic electronic mixing and internal conversion become allowed.

On the other hand, pump-probe spectroscopy after *coherent excitation* of the same set of Cars in polar solvent identified three stimulated-emission components, generated by the quantum-beat mechanism, consisting of the long-lived coherent cross term from the 1B_u_^+^ + 1B_u_^−^ or 1B_u_^+^ + 3A_g_^−^ diabatic pair and incoherent short-lived 1B_u_^+^ and 1B_u_^−^ or 3A_g_^−^ split incoherent terms. The same type of stimulated-emission components were identified in Cars bound to LH2 complexes, their lifetimes being substantially shortened by the Car-to-BChl singlet-energy transfer. The low-energy shifts of the 1B_u_^+^(0), 1B_u_^−^(0) and 3A_g_^−^(0) levels and efficient triplet generation were also found.

Therefore, there is a good chance that not only the 1B_u_^−^ state but also the 3A_g_^−^ state play the role of light-harvesting in bacterial photosynthesis.

In all the above excited-state dynamics, the symmetry properties of the 1B_u_^+^, 1B_u_^−^ and 3A_g_^−^ counterparts are totally conserved during the formation of the diabatic pairs and also during their splitting and relaxation of the 1B_u_^+^ counterpart through emission and the 1B_u_^−^ or 3A_g_^−^ counterpart through internal conversion. This is exactly what has been anticipated by the theoretical description (experimental condition) of the diabatic pairs.

The observed energetics and excited-state dynamics of the diabatic pairs and their rigorous theoretical description using the diabatic basis set fully support the symmetry notations, the energy diagrams and the potential curves for all the 1B_u_^+^, 1B_u_^−^, 3A_g_^−^ and 2A_g_^−^ vibronic levels we have been proposing.

## 7. Relevant Works by Other Investigators

After our proposal of the 1B_u_^−^ and 3A_g_^−^ states, a variety of hidden states have been proposed between the optically-allowed 1B_u_^+^(S_2_) and the optically-forbidden 2A_g_^−^ (S_1_) states. They include the S^*^ [23–25], S*_x_* [26], and S^‡^ [27] states; brief comments on them are given in Ref. [1]. The readers of the present article are encouraged to study the following representative reviews to understand all these proposals in detail and the situation that they are still being debated:

A comprehensive and elaborate review has been published by Polivka and Sundström [28]. These authors studied a large number of publications (more than 330), and introduced all the proposals carefully and equally. Researchers in the field of excited-state energetics and dynamics of Cars can benefit from this excellent review to get a reliable overview. Most recently, these authors published another up-to-date review [29], where a detailed discussion on the 1B_u_^−^ and S^*^ states is given. Despite of self-sacrificing and pains-taking effort, these authors had to conclude that the present proposals are still controversial.

An interesting review, based on femtosecond pump-probe electronic-absorption and subpicosecond stimulated-Raman spectroscopy, was published by Hashimoto, Yoshizawa, De Silvestri and Cogdell [30]. Most recently, Hashimoto, Sugizaki and Cogdell published another review, in which the usefulness of four-wave-mixing spectroscopy is emphasized [31].

The present readers also need to understand what are written in this article is just along an attempt how far the present authors can proceed based on the following simple and systematic picture:

As described in Section 1 (Introduction), the present authors introduced the symmetry notation, 1B_u_^−^ and 3A_g_^−^, based on the Pariser-Parr-Pople calculations done by Tavan and Schulten, including multi-reference double configurational interaction (PPP-MRDCI) [4]. The slopes of the linear dependence of the state energies for what they latter named ‘the 2A_g_^−^, 1B_u_^−^ and 3A_g_^−^ states’, in the ratio of 2:3.1:3.8 determined by the measurement of resonance-Raman excitation profiles for Cars (*n* = 9–13) [1] were in excellent agreement with those theoretically predicted by Tavan and Schulten for shorter polyenes (*n* = 5–8), *i.e.,* 2:3.1:3.7 [4]. In the analysis of the singlet internal conversion and singlet-to-triplet conversion within Car as well as the Car-to-BChl singlet energy transfer, the present authors just used the energy diagram thus obtained (see Figure 1 in Section 1). Here, in this article, they keep using the same notation and report their most recent new findings concerning the unique excited-state properties of the overlapped, optically-allowed 1B_u_^+^ and the optically-forbidden 1B_u_^−^ or 3A_g_^−^ vibronic levels. They have found that just introducing the concept of diabatic interaction to the potential functions of the four low-lying singlet states (1B_u_^+^, 2A_g_^−^, 1B_u_^−^ and 3A_g_^−^), they determined spectroscopically, was enough to explain the excited-state properties of the overlapped vibronic pairs.

To the best of the present authors’ knowledge, no corresponding work has been published. Therefore, it is still immature to write ‘a review article’. However, the motivation to publish ‘a summary’ of our most recent work is as follows: (1) The overlap of the vibrational ladders between the optically-allowed 1B_u_^+^ and optically-forbidden 1B_u_^−^ or 3A_g_^−^ state is an intrinsic property of all-*trans* Cars. (2) Importantly, the diabatic pair, 1B_u_^+^ + 1B_u_^−^ or 1B_u_^+^ + 3A_g_^−^, exhibit sometimes the 1B_u_^−^ or 3A_g_^−^ property, in addition to the 1B_u_^+^ property, depending on (a) the way of pulsed excitation (selective excitation with subpicosecond pulses or coherent excitation with femtosecond pulses), (b) the 1B_u_^+^ vibrational level of initial excitation (the lowest couple or much higher), and (c) the environment of the Car molecule (nonpolar or polar). (3) Therefore, if a laser spectroscopist of Cars were not aware of the phenomena and the mechanisms described in this article, there is a good chance he/she could become confused and introduce additional ‘controversy’ to this field. Therefore, the present authors really would like the above-mentioned leaders as well as relevant colleagues, in this particular field of Car excited states, to carefully read this summary.

We suspect that the following figures, in this article, may be useful to solve the controversy already pointed out by Polivka and Sundström [28,29]: (i) Figures 10 and 11 showing the diabatic electronic mixing in the 1B_u_^+^ + 1B_u_^−^ pair, after selective excitation with 100 fs pulses in nonpolar solvent, as well as Figures 19 and 21 showing the quantum beat of the 1B_u_^+^ + 1B_u_^−^ pair, after coherent excitation with 30 fs pulses in polar solvent. They must help to understand the subtle 1B_u_^+^ and 1B_u_^−^ transition dipole moments. Rather confusing ‘1B_u_^+^’ and ‘1B_u_^−^’ lifetimes may also depend on such experimental conditions. (ii) Figure 24 (components 2 to 3 in the top-left panels) showing transformation from the 1B_u_^−^ state into the 2A_g_^−^ and T_1_ states; the former is due to singlet internal conversion, while the latter is due to singlet-to-triplet fission. If the S^*^ became time-resolved into the 1B_u_ and T_1_ (1^3^B_u_) components, the contradiction between the 1B_u_^−^ and S^*^ states should be solved. (iii) Figure 10 (top-left two panels) showing the generation of a diabatic pair and Figure 30 (top two panels) showing the splitting of the diabatic pair. The apparent 1B_u_^+^ lifetime depends on the excited vibrational level in the 1B_u_^+^ manifold (bottom or higher).

Here, in the rest of this section, we will describe the results of *ab initio* calculations including both σ and π electrons [32] (Section 5.1) and the observation of oscillatory intensity changes immediately after pulsed excitation to the 1B_u_^+^ state of lutein, *i.e.*, Car (*n* = 10) [33,34] (Section 5.2). We think these two topics are relevant to our present findings.

### 7.1. Ab Initio Calculation of the π → π* Excited States of Linear Polyenes [32]

In this theoretical paper, multi-reference Møller-Plesset perturbation theory with complete active-space configurational interaction (CASCI-MRMP) was applied to calculate the energies of the vertical π → π* transitions of all-*trans* polyenes (*n* = 3–14), focusing on the nature of the four lowest-lying singlet-excited states and their ordering: It has been determined that the ionic 1B_u_^+^ state is the lowest optically-allowed excited state, while the covalent 2A_g_^−^, 1B_u_^−^ and 3A_g_^−^ states are the optically-forbidden states increasing in energy in this order. The calculations predict that the 1B_u_^−^ state becomes lower than the 1B_u_^+^ state at *n* ≥ 7, while the 3A_g_^−^ state becomes lower than the 1B_u_^+^ state at *n* ≥ 11.

It was a challenge for the theoreticians to carry out highly accurate *ab initio* calculations of the longer polyenes to realize the state ordering determined by the use of resonance-Raman excitation profiles. Before starting the calculations, the authors had demonstrated (in other molecular systems) that CASCI-MRMP was more efficient than, and comparable in accuracy to MRMP based on CASSCF reference functions.

To calculate the vertical excitation energies from the ground state to the relevant singlet states, the ground-state equilibrium geometries were optimized at the MP2 level. A reference CASCI wave function was obtained by partitioning the SCF orbitals, and optimizing the expansion coefficients of all configurations that were generated by all the possible arrangement of the active electrons among the active orbitals. The 10 valence π electrons were treated as active electrons. The effect of σ electrons was included through the perturbation calculation performed with MRMP, which was applied to each individual excited state.

In the present case of alternant hydrocarbons, the pairing properties are satisfied at the CASSCF and even the CAS-CI level. The 1A_g_^−^, 1B_u_^+^, 2A_g_^−^, 1B_u_^−^ and 3A_g_^−^ states could be characterized by the use of it.

The calculated vertical-excitation energies did not exhibit a simple linear dependence on 1/(2*n +* 1) but fit to an exponential function, for fixed *n*_0_,

$$E_{n}=E_{\infty }+(E_{n0}-E_{\infty })\;\text{exp}\;[-a\;(n-n_{0})]$$

**Figure 31.**
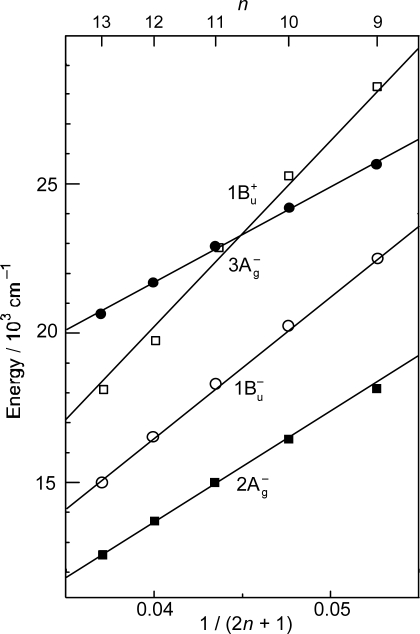
The energies of the optically-allowed 1B_u_^+^ and the optically-forbidden 2A_g_^−^, 1B_u_^−^ and 3A_g_^−^ states (for vertical transition from the ground 1A_g_^−^ state) that have been obtained by *ab initio* calculations including the σ- and π-electrons by the use of Møller-Plesset perturbation theory with complete active-space configurational interaction (CASCI-MRMP). Approximate linear relations, as functions of 1/(2*n* + 1), are presented for comparison with Figure 1 (Reproduced from Ref. [32]).

Figure 31 shows an energy diagram for *n* = 9–13; approximate linear regression lines, as functions of 1/(2*n* + 1), are drawn for comparison to those in Figure 1. The state ordering of the four lowest-lying singlet states, *i.e.*, 1B_u_^+^, 2A_g_^−^, 1B_u_^−^ and 3A_g_^−^, determined by the measurement of resonance-Raman excitation profiles (Figure 1) is in general agreement with that predicted by the highly accurate *ab initio* calculations including the σ- and π-electrons (Figure 31). The absolute values of the state energies and their dependence on *n* are still in poor agreement. When agreement between the observed and calculated state ordering becomes closer in the future, the results of calculations must provide us with a most reliable clue to confirm the symmetry notation of each singlet-excited state. It is much more straightforward to determine the symmetry of each excited state theoretically rather than spectroscopically, although the latter is ideal.

### 7.2. Oscillatory Intensity Changes in Electronic Absorption Immediately after Pulsed Excitation of Lutein [33,34]

**Figure 32.**
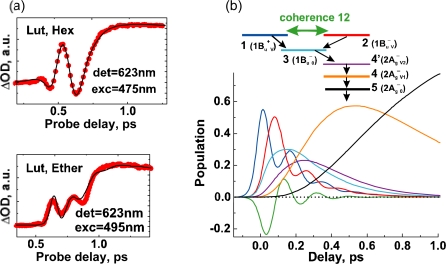
Quantum beat due to coherence coupling between the 1B_u_^+^ and 1B_u_^−^ vibronic levels in lutein, *i.e.*, Car (*n* = 10): **(a)** The intensity fluctuations of transient absorption were most pronounced in the 600–700 nm region. **(b)** The intensity fluctuations simulated by a scheme consisting of 5 components, whose changes in population are shown below (Reproduced from Ref. [34]).

Following a preliminary report on *β*-carotene [33], Alfred Holzwarth and his coworkers have most recently reported the intensity fluctuation of transient absorption immediately after the excitation of lutein, *i.e.*, Car (*n* = 10), to the 1B_u_^+^ state and ascribed it to quantum beat due to coherence coupling between the 1B_u_^+^ and 1B_u_^−^ vibronic levels [34]. Oscillatory intensity changes were most pronounced in the 600–700 nm region as shown in Figure 32a. The oscillatory changes in the electronic absorption were simulated by a scheme consisting of five components, whose time-dependent changes in population are shown in Figure 32b. The results of simulation are overlaid on the time profiles in Figure 32a. Interestingly, the profile and magnitude of intensity changes were strongly dependent on the solvent, which probably reflect the shift of the 1B_u_^+^ counterpart in energy. Also, they are strongly dependent on the wavelengths of excitation and detection.

**Figure 33.**
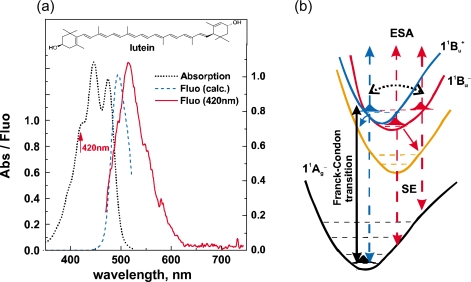
**(a)** Asymmetric fluorescence pattern consisting of the overlapped 1B_u_^+^ and 1B_u_^−^ fluorescence and **(b)** the 1B_u_^+^ and 1B_u_^−^ potential curves obtained by theoretical calculation based on (a). ESA and SE stand for excited-state absorption and stimulated emission (Reproduced from Ref. [34]).

To prove that the counterpart of the 1B_u_^+^ state is the 1B_u_^−^ state in the coherence coupling, the fluorescence pattern that does not exhibit the mirror image with respect to the electronic-absorption pattern (as shown in Figure 33a) was theoretically analyzed in lutein and *β*-carotene. The 1B_u_^+^ and 1B_u_^−^ energies and potentials have been *theoretically* determined as shown in Figure 33b. Most importantly, the pair of the 1B_u_^+^ and 1B_u_^−^ energy levels is almost iso-energetic and located close to the conical intersection, showing the reliability of their theoretical calculations.

The above results of analysis and interpretation in the case of *β*-carotene and lutein seem to be closely correlated to our case of bacterial Cars. In our terminology, it may be called ‘coherent excitation of the 1B_u_^+^ + 1B_u_^−^ diabatic pair’ in lutein.

It is also encouraging, that these authors proved the presence of the 1B_u_^−^ state, which is coherently coupled with the 1B_u_^+^ state. Detailed discussion on the nature of the 1B_u_^−^ state and the mechanism of quantum beat based on their sophisticated theoretical calculations are described in their original literature. Such theoretical analysis combined with spectroscopic studies will reveal the more detailed mechanisms of diabatic electronic mixing and quantum beat.

## Figures and Tables

**Table 1.** Dependence of the 1B_u_^+^, 1B_u_^−^, 3A_g_^−^ and 2A_g_^−^ lifetimes (in ps) on the number of conjugated double bonds (*n*) (Reproduced from Ref. [12]).

	Neurosporene (*n* = 9)	Spheroidene (*n* = 10)	Lycopene (*n* = 11)	Anhydrorhodovibrin (*n* = 12)	Spirilloxanthin (*n* = 13)
1B_u_^+^	0.10	0.10	0.02	0.01	0.01
1B_u_^−^	0.24	0.23	–	–	–
3A_g_^−^	–	–	0.15	0.10	0.10
2A_g_^−^	24.0	8.9	3.9	2.2	1.4
